# PSMA-targeted theranostic nanoplatform achieves spatiotemporally precise therapy and triggers ferroptosis in prostate cancer treatment

**DOI:** 10.1186/s13046-025-03530-4

**Published:** 2025-09-30

**Authors:** Linxue Zhang, Qi Sun, Dongxin Zheng, Xiang Huang, Zhong Yu, Zhongwen Lan, Wei Xiong, Ke Sun, Ruiji Liu

**Affiliations:** 1 School of Materials and Energy, University of Electronic Science and Technology of China, Chengdu, 610054 P. R. China; 2 Department of Urology, School of Medicine, Sichuan Provincial People’s Hospital, University of Electronic Science and Technology of China, Chengdu, 610072 China

**Keywords:** Theranostics, Magnetic hyperthermia therapy, PSMA targeting, SiRNA delivery, Ferroptosis

## Abstract

**Background:**

Ferroptosis, an iron-dependent form of regulated cell death, is crucial for the fate of tumors such as prostate cancer (PCa) under conditions of metabolic and oxidative stress. Consequently, the disruption of ferroptosis defense mechanisms could be lethal to these cancer cells, while sparing normal cells. Despite this potential, the development of effective and controlled in vivo therapies targeting ferroptosis remains underexplored.

**Methods:**

In this study, liposomes modified with Glu-urea-Lys (GUL) encapsulating Mn_0.6_Zn_0.4_Fe_2_O_4_ (MZ) were employed as siRNA delivery vectors targeting Ying Yang 1 (YY1) for PCa treatment both in vitro and in vivo. The synergistic antitumor effects of the GUL@LsiYY1@MZ nanosystem were assessed using CCK8 assays, Western blot analysis, flow cytometry, and laser scanning confocal microscopy imaging in vitro. Additionally, the mechanisms underlying the ferroptosis effects were further explored through transcriptome and lipidomics sequencing. Intravenous administration was employed to treat subcutaneous tumors in a mouse model, and the tumor inhibitory effects, safety, and visibility on T_2_-weighted MRI were evaluated.

**Results:**

The engineered GUL@LsiYY1@MZ nanosystem exploits the specific binding affinity of GUL for the prostate membrane-specific antigen (PSMA), facilitating targeted delivery and accumulation. Upon exposure to alternating magnetic fields (AMF), this system enables the precision-controlled release of siRNA into the cell, leading to the knockdown of YY1 expression. This downregulation subsequently affects the expression of the SLC7A11, thereby disrupting glutathione metabolism. Additionally, the introduction of excess Fe^2+^ induces iron overload, further promoting ferroptosis. Significantly, this therapeutic intervention restructured the metabolism of PCa cells, leading to a substantial intracellular accumulation of unsaturated fatty acids. This accumulation provided an abundant substrate for the generation of phospholipid peroxides, ultimately compromising plasma membrane integrity and inducing ferroptosis in PCa cells. Furthermore, the nanosystem also functions as a contrast agent, enhancing the T_2_-weighted MRI imaging of solid tumors.

**Conclusion:**

The GUL@LsiYY1@MZ nanosystem utilizes AMF-triggered release to downregulate SLC7A11, inducing ferroptosis and contributing to enhanced anti-tumor efficacy.

**Graphical abstract:**

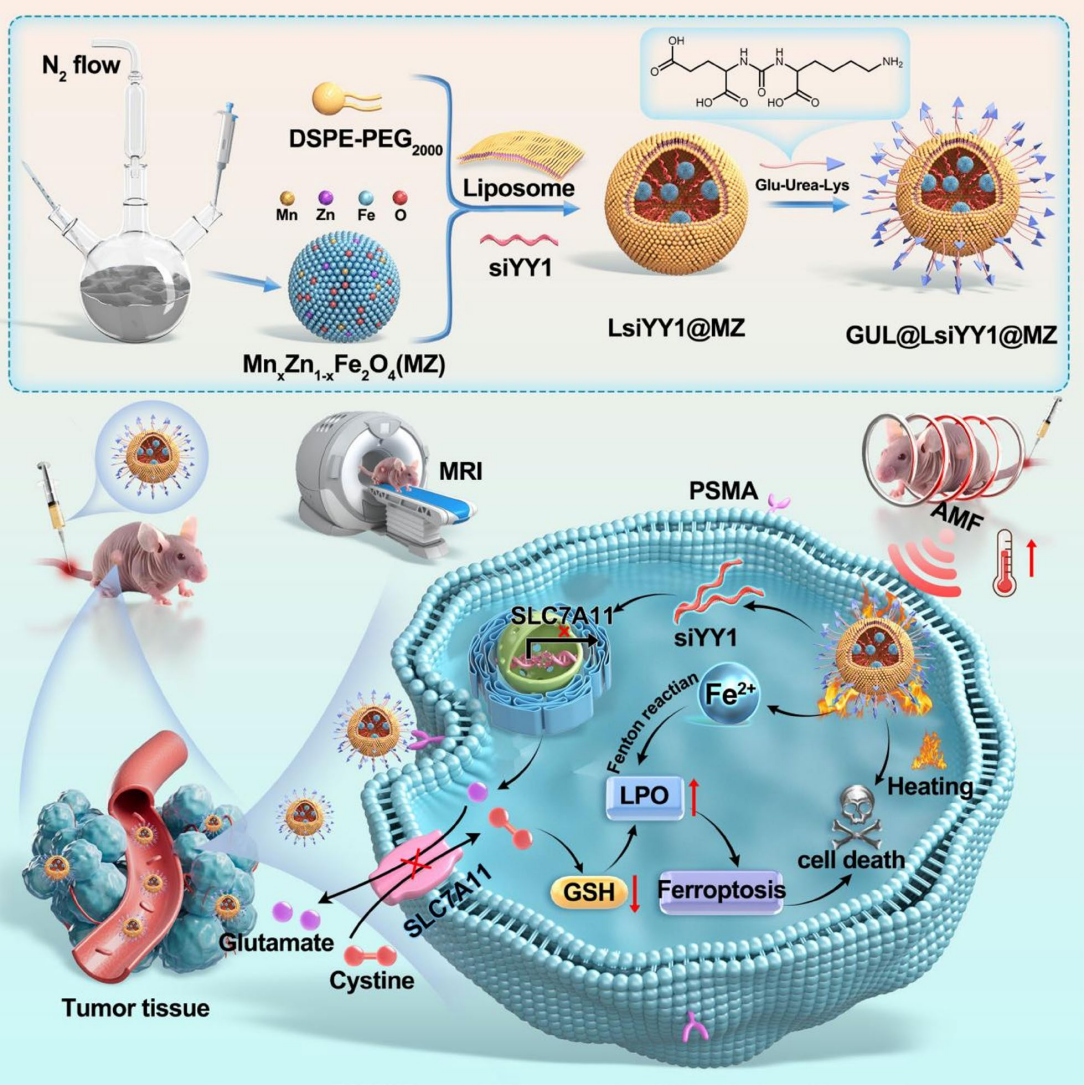

**Supplementary Information:**

The online version contains supplementary material available at 10.1186/s13046-025-03530-4.

## Introduction

Prostate cancer (PCa) remains the most prevalent malignancy in men worldwide, with an estimated 299,010 new cases and 35,250 deaths projected in the United States for 2024 [1]. While radical prostatectomy offers a curative option for localized tumors, its utility is limited by patient comorbidities and advanced disease stages [2]. Androgen deprivation therapy (ADT), the frontline treatment for metastatic PCa, initially controls tumor growth but inevitably fails as tumors evolve into castration-resistant prostate cancer (CRPC) - a lethal phase marked by therapeutic resistance, metastasis, and poor survival [3]. This transition is driven by molecular adaptations, including the overexpression of transcription factor Yin Yang 1 (YY1), which promotes tumor plasticity and survival under ADT pressure. Despite advances in CRPC management, therapies targeting these adaptive mechanisms remain scarce, underscoring the urgent need for innovative strategies.

Ferroptosis is an iron-dependent form of regulated cell death characterized by the toxic accumulation of lipid peroxides on the cell membrane, resulting in impaired plasma membrane permeability and ultimately cell demise [4, 5]. In recent years, significant advancements have been made in elucidating the role of ferroptosis in tumor biology and therapy. On one hand, the evasion of ferroptosis mediated by oncogenes or oncogenic signaling pathways contributes to tumorigenesis, progression, metastasis, and treatment resistance [6, 7]. Conversely, the distinct metabolism, elevated levels of reactive oxygen species (ROS), and specific mutations in cancer cells render some of them inherently susceptible to ferroptosis, thereby revealing the vulnerability of certain cancer types [8]. Recent research has demonstrated that glutamine metabolism is consistently upregulated in CRPC tumors, and this enhanced glutamine metabolism drives an antioxidant program that enables tumor cells to tolerate higher basal levels of ROS, thereby promoting the growth of enzalutamide-resistant prostate cancer [9]. Consequently, CRPC appears to be particularly reliant on ferroptosis mechanisms of resistance to survive under conditions of metabolic and oxidative stress. Thus, disruption of these defenses would be lethal to these cancer cells, whereas normal cells would be unaffected.

YY1, a multifunctional transcription factor regulating ∼ 10% of human genes [10], is aberrantly overexpressed in aggressive PCa and correlates with poor prognosis [11]. Our prior work demonstrated that YY1 drives CRPC progression by enabling metabolic reprogramming and treatment resistance [12]. Silencing YY1 via siRNA not only suppresses tumor proliferation and metastasis in vitro but also delays subcutaneous tumor growth in vivo, positioning it as a compelling therapeutic target. Furthermore, recent research has revealed that YY1 extensively inhibits ferroptosis across various cancers by augmenting antioxidant pathways, such as SLC7A11-mediated glutathione synthesis [13] and PLK1-driven NADPH production [14]. This activity promotes tumor survival under metabolic and therapeutic stress, positioning YY1 as a potential target for inducing ferroptosis in the treatment of refractory tumors.

However, siRNA delivery faces critical barriers: poor cellular uptake, enzymatic degradation, and inefficient endosomal escape. Liposomes nanoparticles (LNPs) have emerged as promising siRNA carriers due to their biocompatibility, high loading capacity, and endosomal escape capabilities [15]. However, standalone LNPs lack spatiotemporal control over siRNA release and fail to address tumor heterogeneity. Magnetic hyperthermia therapy (MHT), which utilizes alternating magnetic fields (AMF) to activate iron oxide nanoparticles (MNPs), offers a solution by enabling localized heat generation (42–45 °C) for triggered drug release and direct tumor ablation [16]. Despite these advantages, conventional MNPs suffer from low magnetothermal efficiency and off-target toxicity, limiting their clinical translation [17].

To overcome these challenges, we have designed GUL@LsiYY1@MZ - a temperature-sensitive magnetic lipid nanohybrid that combines Mn_0.6_Zn_0.4_Fe_2_O_4_ (MZ) nanoparticles with liposomes loaded with YY1-targeting siRNA, conjugates of small molecules targeting PSMA (Glu-urea-Lys, GUL) [18]. The platform uniquely integrates four features: (1) MRI-guided tumor localization via T_2_-weighted contrast, (2) Targeted recognition of PSMA-positive prostate cancer cells by Glu-urea-Lys. (3) AMF-triggered siRNA release at mild hyperthermia, and (4) induction of ferroptosis by inhibition of SLC7A11. Mn-Zn doping enhances MZ’s magnetothermal efficiency beyond prior reports [19, 20]. GUL efficiently recognizes PSMA and is only taken up by PSMA positive PCa cells, ensuring precise targeting of this delivery platform, while thermosensitive liposomes ensure siRNA protection and on-demand release. By synchronizing gene silencing with iron-dependent lipid peroxidation, GUL@LsiYY1@MZ disrupts redox homeostasis in CRPC cells, offering a dual-mode therapeutic strategy against treatment-resistant tumors.

## Materials and methods

### Bioinformatics analysis of scRNA-seq data

The scRNA-seq data of enzalutamide-resistant prostate cancer (GSE168668) were obtained from the GEO database (https://www.ncbi.nlm.nih.gov/geo/) [21]. In summary, the Seurat package was employed to create objects and exclude low-quality cells, while standard data preprocessing techniques were applied. To normalize the library size effect in each cell, we scaled UMI counts using scale.factor = 10,000. The corrected-normalized data metrics were applied to the standard analysis as described in the Seurat R package. The top 2,000 variable genes were extracted for principal component analysis (PCA). The top 15 principal components were kept for UMAP visualization and clustering [22]. Subsequently, the AverageExpression function was utilized to compute the mean expression value of cells within each subgroup. The pathway scores for each cell subgroup or group were then calculated using the GSVA package, and the results were visualized using pheatmap.

### Materials

Zinc acetylacetonate (Zn(acac)_2_), Manganese acetylacetonate (Mn(acac)_2_), Ferric acetylacetonate (Fe(acac)_3_), chloroform, 1-octadecene, toluene, oleyamine, dimethyl sulfoxide (DMSO), hexane and oleic acid (OA) were obtained from Sigma-Aldrich (USA). SiRNA-YY1-cy5 were designed by Co., Ltd GenePharma (China). 1,2 - distearoyl - sn - glycerol − 3 - phosphoethanolamine - N - [methoxy (polyethylene glycol)-2000] (DSPE-mPEG_2000_), phosphatidylcholine and cholesterol were obtained from Aoshuo Co., Ltd (China). 1-Ethyl-3-(3-dimethylaminopropyl) carbodiimide (EDC) and N-Hydroxysuccinimide (NHS) were purchased by Sigma (USA). Glu-urea-Lys (GUL) was obtained from Targetmol Co., Ltd (USA). BODIPY 581/591 C11 and FerroOrange were purchased by MedChemExpress (USA) and Dojindo (Japan), respectively. ROS assay kit was purchased by Thermo Fisher Scientific Co., Ltd (USA). Ultrapure water and deionized water were used in all the experiments. All siRNA-related experiments in this work utilized diethyl pyrocarbonate (DEPC) water act as protective solvent.

### Synthesis of GUL@LsiYY1@MZ nanohybrids

#### Synthesis of ultrasmall Mn_x_Zn_1–x_F

Monodisperse Mn_x_Zn_1–x_F NPs (x = 0.1, 0.2, 0.4, 0.6, 0.7, 0.8) were synthesized by with high temperature thermal decomposition as a previous method [23]. In brief, different content of Zn(acac)_2_, Mn(acac)_2_ and Fe(acac)_3_ under N_2_ condition and stirred continuously in mixture of oleyamine, OA and 1-octadecene. A summary of the detailed reaction parameters is shown in Table S1. The above solution was reacted at 200 °C degrees for 2 h. Subsequently, the resulting solution was heated to 300 °C for 1 h. The resulting Mn_x_Zn_1–x_F NPs were collected by centrifugation at 10,000 rpm for 15 min at 25℃. This step was repeated twice to ensure complete separation. The Mn_x_Zn_1–x_F NPs were washed three times with a 1:5 (v/v) mixture of ethanol and toluene to remove organic residues. After each wash, the particles were redispersed via sonication (5 min, 25 °C) and centrifuged again under the above conditions. After comprehensive analysis of magnetothermal properties, the Mn_0.6_Zn_0.4_F was selected for the following research.

#### Preparation of liposomes

Liposomes were prepared using the typically thin-film hydration method, followed by extrusion for refinement [24]. Phosphatidylcholine, cholesterol and DSPE-PEG_2000_ were dissolved in chloroform. The solvent was then removed using a rotary evaporator to form a uniform lipid film. After drying the film, it was hydrated with PBS buffer to generate a liposome precursor solution. Subsequently, the liposomes were formed by employing sonication.

#### Synthesis of thermosensitivity LsiYY1@MZ

MZ NPs and siRNA were co-encapsulated into liposomes. First, MZ NPs were modified by DSPE-PEG_2000_. Next, ammonia was added into above mixture under vigorous stirring for overnight [25]. Subsequently, solvents in above solution were removed by evaporation in a fume hood. The above mixtures were sterilized at high temperature. DEPC water was introduced to dissolve the remaining substances.

Next, siRNA solution (50 nM) was prepared and dissolved in DEPC. The siRNA and MnZn ferrite were gradually added to the liposome precursor solution, with gentle mixing to ensure even distribution. Then the mixed solution was placed in ultrasound to promote the formation of liposomes. The unencapsulated components were removed by ultrafiltration to obtain the purified liposome hybrids. After the reaction, part of the solvent was evaporated under reduced pressure at room temperature, and the remaining concentrated solution was sucked into a centrifuge tube, and an appropriate amount of cyclohexane was added for centrifugation. The supernatant was discarded, and the operation was repeated twice. The solid obtained by centrifugation was placed at 35 ◦C for 30 min under reduced pressure to obtain target product LsiYY1@MZ. The product was dispersed in 3 mL of DEPC solution. The obtained LsiYY1@MZ nanohybrids were kept at 4 °C.

#### Construction of GUL@LsiYY1@MZ hybrids

LsiYY1@MZ nanoparticles (10 mg) were added to 2 mL of DEPC solution. Then, 1 mL of freshly prepared 10 mM EDC solution was added, and the mixture was thoroughly mixed for 30 min. Next, 1 mL of freshly prepared 10 mM NHS solution and 1 mg of Glu-urea-Lys were added. After ensuring uniform mixing, 1 mL of 0.8 mM ethylenediamine was introduced. The reaction was carried out on a shaker at room temperature for 8 h. The GUL@LsiYY1@MZ product was dialyzed for 72 h using a 3 kDa dialysis membrane to remove unreacted small molecules.

### Characterization of MZ nanoparticles and GUL@LsiYY1@MZ

The morphology and size distribution of Mn_x_Zn_1–x_F nanoparticles and GUL@LsiYY1@MZ hybrids were recorded by transmission electron microscopy (TEM) (Tecnai G2 F20 S-Twin TMP). Both in vitro and in vivo experiments quantified the Fe concentration in the GUL@LsiYY1@MZ nanocomposite. Thermogravimetric analysis (TGA) was determined by Mettler toledo. The UV-Vis spectrum of the samples was determined by UV-vis spectrophotometer (PerkinElmer Inc). All the samples value of hydrodynamic diameters and zeta potential were recorded by Zeta Plus analyzer. The magnetic properties of the samples were measured by vibrating sample magnetometer instruments (VSM, Lake shore 8604). An infrared imager (Fotric 343) was used to record the temperature changes of the samples. MIS Hyperthermia System was used to measure the magneto-thermal conversion capacity of the samples (324 Oe, 270 kHz).

### Temperature-Dependent SiRNA release from GUL@LsiYY1@MZ

A 1.0 mg/mL aqueous suspension of GUL@LsiYY1@MZ at different temperatures (treated with 37, 39, 41, and 43 ℃) was centrifuged at 15,000 rpm for 10 min, and the supernatant was analyzed for absorbance at 260 nm. The siRNA release percentage was determined by comparing the released siRNA amount to the total encapsulated siRNA.

### Cell culture and cellular uptake

The Human prostate cancer cell lines LNCaP, C4-2 and PC3 were purchased from American Type Culture Collection (Manassas, VA, USA), and cultured in RPMI 1640 (Gibco) containing 10% FBS (Gibco) and penicillin-streptomycin (2%) at 37 ℃ with 5% CO_2_ in cell incubator. LNCaP and C4-2 cells were seeded at 2 × 10^5^ cells per well in a 6-well plate. When the cell density reached approximately 70–80%, cells in each well was treated with different treatments, including pure MZ and GUL@LsiYY1@MZ (untreated cells as control group). After 24 h of incubation in cell incubator, the cells were treated with/without a magnetic field (15 min, 324 Oe, 270 kHz) according to the respective treatment five groups: control, MZ, GUL@LsiYY1@MZ, MZ + AMF and GUL@LsiYY1@MZ + AMF.

### Cell viability assay

LNCaP and C4-2 cells were cultured in six-well plates until reaching a confluence of 60–70%. At this point, various nanoparticles were introduced, and the cells were incubated for an additional 6 h. This was followed by a 15-minute exposure to an AMF with parameters set at 324 Oe and 270 kHz. Subsequently, the culture dishes were rinsed with PBS and replenished with fresh culture medium, allowing for further incubation over an 18-hour period. Cells were then harvested and reseeded into 96-well plates. After a 24-hour incubation period, cell viability was evaluated using the Cell Counting Kit-8 (CCK-8, Beyotime). Absorbance at 450 nm was measured to quantify cell viability [26]. The CCK-8 assays were conducted in five independent experiments (*n* = 5).

### Detection of in vitro ROS, LPO and Fe ^2+^ generation

The cells were seeded into confocal dishes (biosharp) (density: 3 × 10^5^ cells per well) for 24 h in incubator. Control, MZ, GUL@LsiYY1@MZ, MZ + AMF and GUL@LsiYY1@MZ + AMF were administrated to each group and cultured for 48 h, the cells in the above treatment group were placed in a magnetic field for 15 min. Then, the medium was removed and wash three times used PBS. The FBS-free medium was then mixed with FerroOrange, DCFH-DA and BODIPY, respectively. The cellular uptake and fluorescent images of the cells were visualized and analyzed by laser scanning confocal microscopy (LSM800, ZEISS, Germany). Correspondingly, flow cytometry was used to quantitatively detect the production of intracellular ROS, LPO and Fe ^2+^. The cells were seeded into 6-well plates (density: 2 × 10^5^ cells per well) according to the above five groups. The concentration of MZ and GUL@LsiYY1@MZ was 100 µg/ml. After 48 h of incubation, the medium was removed and added fresh medium without FBS. FerroOrange (Fe ^2+^ probe, 1 µM), DCFH-DA (ROS probe, 5 µM) and C11-BODIPY (Lipid ROS probe, 5 µM) were applied in different group and incubated for 30 min into 6-well plates. Then the adherent cells were digested with trypsin, centrifuged, resuspended with 0.5 mL of PBS, and transferred to flow tubes to test the fluorescence intensity via flow cytometry (Quanteon). All measurements were conducted in three independent experiments (*n* = 3).

### Western blotting assay of related proteins

Western blot analysis involved lysing the treated cells with RIPA buffer (Yamei, China), and then in a cell crusher to further shatter the cells (on ice). The lysates were obtained by centrifuging at 15,000 rpm for 30 min at 4 °C. After the protein content is determined by BCA method, the protein sample is put into the protein boiler after loading adjustment (100 °C, 5 min). The protein was then subjected to SDS-PAGE gels and transferred onto PVDF membranes. SLC7A11 polyclonal antibody (Thermo Fisher, USA, 1:1000) and YY1 polyclonal antibody (CST, USA, 1:4000) incubated overnight at 4 °C. Following an additional incubation with goat anti-Rabbit IgG cross-adsorbed secondary antibody (Thermo Fisher, USA, 1:5000) for 1 h at room temperature. The bands were visualized using the Imaging system (Bio-Rad).

### MR imaging performance

MRI was performed by a clinical 3T MR imaging device (Discovery MR758, 3.0T) in which the tubes were vertically placed inside an animal coil. T_1_ weighted imaging measurements: an SE sequence with TR = 350 ms, TE = 4.5 ms. T_2_ weighted imaging measurements: an SE sequence with TE = 2000 ms, TE = 40 ms. According to a previous study [27], T_1_ relaxation measurements, an SE-IR sequence with increasing inversion times (T_I_ = 50, 400, 1100 and 2500 ms) and a fixed TR of 2600 ms. T_2_ relaxation measurements was conducted using a multi-echo spin-echo sequence with TE ranging from 10 to 80 ms and a fixed TR of 2000 ms. Additional imaging parameters included a 1.5 mm slice thicknes, 128 × 128 matrix, and average = 4. The r_1_ and r_2_ relaxivity were calculated from the slope of the linear regression between 1/T_1_ or 1/T_2_ and the corresponding Fe concentration (0.1, 0.2, 0.4, 0.8, and 1 mM). LNCaP cells underwent enzymatic digestion with trypsin, followed by centrifugation and two washes with PBS. Subsequently, a cell suspension was prepared at a concentration of 1 × 10^7^ cells per 0.1 mL using RPMI 1640 medium combined with Matrigel in a 1:1 ratio. This suspension was subcutaneously inoculated into the axillary region of BALB/c nude male mice, with each mouse receiving 0.1 mL to establish the LNCaP tumor-bearing mouse model. Once the tumor volumes reached approximately 70 mm^3^ (Tumor volume = 1/2 × Length × Width²), a volume of 200 µL of PBS, LsiYY1@MZ, and GUL@LsiYY1@MZ nanoparticles (at a concentration of 1.5 mg/kg) was administered intravenously to the mice.

### In vivo tumor growth Inhibition study

The mice were randomly divided into five groups (*n* = 5 for each group): PBS, MZ, GUL@LsiYY1@MZ, MZ + AMF and GUL@LsiYY1@MZ + AMF. When the tumor volumes reached approximately 70 mm^3^, the mice were administered 200 µL of PBS, pure MZ and pure GUL@LsiYY1@MZ at a dose of 1.5 mg/kg via intravenous injection (magnetic field guided and stimulation). At the same time, the pure MZ, GUL@LsiYY1@MZ were injected into mice, respectively and exposed to alternating magnetic field for 30 min to obtain MZ + AMF and GUL@LsiYY1@MZ + AMF group. The treatment cycle lasted 14th days, and the plan is as follows: Inject the PBS, pure MZ and pure GUL@LsiYY1@MZ on days 0, 3, 6, 9, and 12, and then after interval of 24 h, place mice that require magnetothermal therapy in MIS Hyperthermia System (Magnetic field: 324 Oe, 270 kHz) for 30 min (MZ + AMF and GUL@LsiYY1@MZ + AMF group). The mice were sacrificed until 14 days treatment.

### Statistical analysis

In this research, all experimental data come from at least three independent experiments. The data are expressed as mean ± standard deviation (SD) and were analyzed utilizing GraphPad Prism 10 software. For comparisons between two groups, the Student’s t-test was employed, while One-way ANOVA with Tukey’s post-hoc test was used for multi-group comparisons. Significance levels: *p < 0.05, **p < 0.01, ***p < 0.001, ****p < 0.0001.

## Results and discussion

### Single-cell sequencing analysis suggested that reactive oxygen species level was up regulated in enzalutamide-resistant prostate cancer

**Fig. 1:**
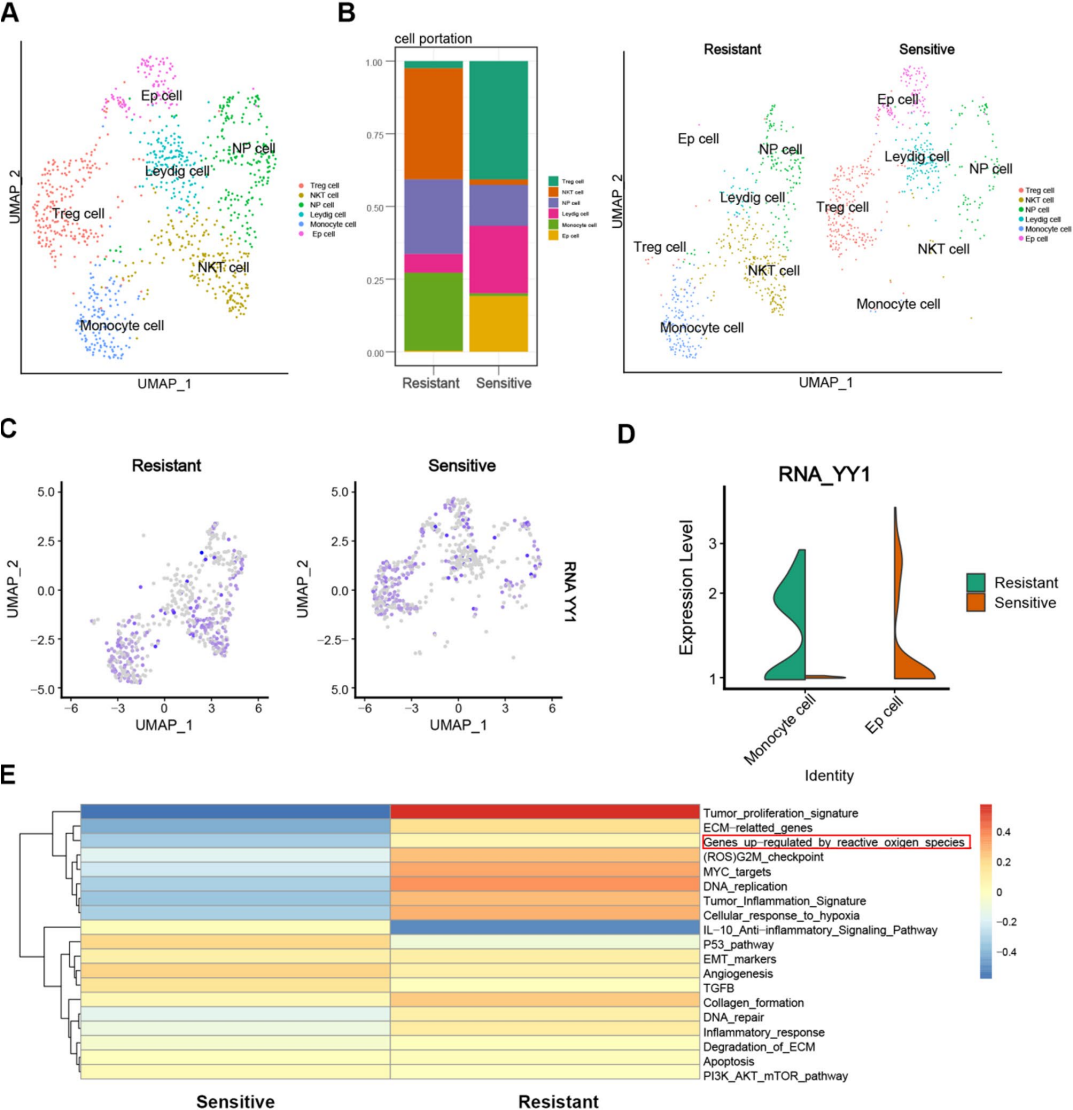
Analyses of the scRNA-seq on the relationship between YY1 expression and enzalutamide-resistance. (**a-b**) UMAP of tumor cells of the GSE168668 dataset annotated by marker genes. (**c-d**) Expression map of YY1 in enzalutamide sensitive and resistant groups. (**e**) Heatmap of enriched pathways for cell subsets

Following the application of the UMAP method for cell grouping, cell populations were annotated using marker genes (Fig. 1A). Our analysis revealed a predominance of NKT cells and monocytes in enzalutamide-resistant prostate cancer, whereas epithelial and Leydig cells were more prevalent in enzalutamide-sensitive cases (Fig. 1B). These observations align with our previous findings, which indicated an upregulation of YY1 in enzalutamide-resistant prostate cancer cells, particularly within monocytes (Fig. 1C, D). Recent research has demonstrated that glutamine metabolism is persistently upregulated in castration-resistant prostate cancer (CRPC) tumors, facilitating an antioxidant program that enables tumor cells to endure elevated basal levels of reactive oxygen species (ROS) [28]. In the current study, we observed an upregulation of the ROS pathway in the enzalutamide-resistant prostate cancer group (Fig. 1E). This suggests that enzalutamide-resistant prostate cancer cells possess a distinctive metabolic defense mechanism that aids their adaptation to the high-ROS environment induced by enzalutamide treatment. Disruption of this metabolic equilibrium often proves detrimental to resistant cells [29]. Consequently, the development of nanomaterials capable of delivering siRNA to prostate cancer cells and targeting YY1 to induce ferroptosis presents a promising avenue for therapeutic exploration.

### Characterization and magnetothermal efficiency of Mn_x_Zn_1−x_F

**Fig. 2:**
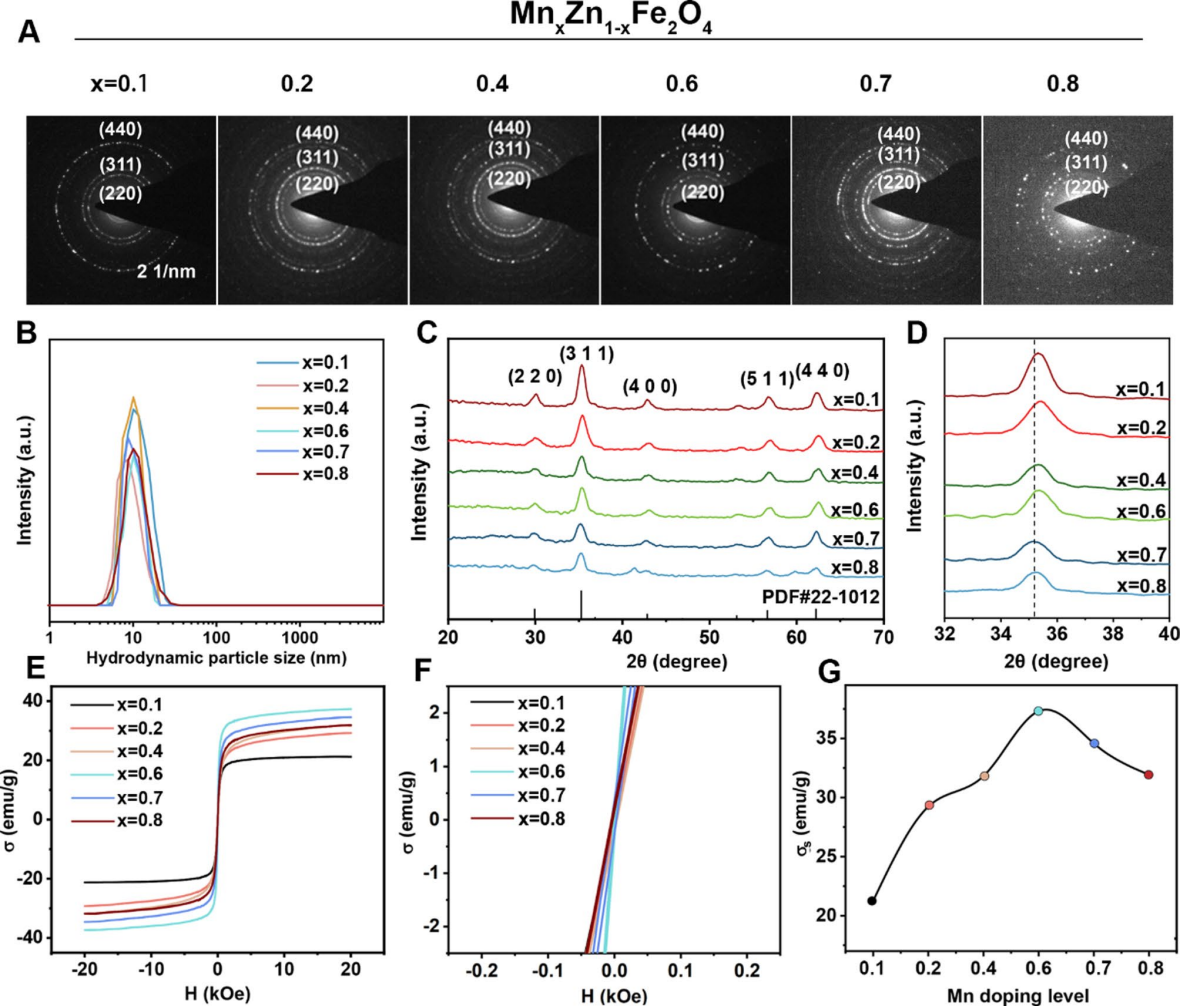
Characterization of nanoparticles with various Mn*/*Zn doping ratios Mn_*x*_Zn_1–*x*_F (*x* = 0.1, 0.2, 0.4, 0.6, 0.7, 0.8). (**a**) SAED imaging of samples. (**b**) Particle sizes. (**c-d**) XRD spectra and enlarged (311) diffraction peaks. (**e-f**) M-H loop at 300 K and magnification image. (**g**) σ_s_ varies with Mn content

Transmission electron microscope (TEM) images of the representative Mn_*x*_Zn_1–*x*_Fe_2_O_4_ (denoted as Mn_x_Zn_1−x_F) (*x* = 0.1, 0.2, 0.4, 0.6, 0.7, 0.8) reveal high monodispersity (Figure S1). A series of Mn_x_Zn_1−x_F nanoparticles with different doping levels were prepared by carefully varying the initial molar ratio of the manganese acetylacetonate and zinc acetylacetonate precursors, the morphology of the Mn_x_Zn_1−x_F did not change obviously. All the as-obtained Mn_x_Zn_1−x_F samples were spherical with uniform size around 9.0 ∼ 10.0 nm, which were indicative of single domain. The high-resolution TEM images of the samples (insert of Figure S1) suggested that the as-prepared nanoparticles possess high crystallinity. The spinel cubic crystalline nature of the magnetic crystallinity could be confirmed by the distinct lattice fringe patterns and selective area electron diffraction (SAED) pattern (Fig. 2A). It was to note the surface disordered structure cannot be observed due to the amorphous carbon grid background and extremely small size of the nanoparticles, whereas it was well-known that, with the decreasing of the size of nanoparticles, the portion of the surface disordered structure will increase. Together, these results confirmed the successful preparation of Mn_x_Zn_1−x_F with different doping levels. Studying the crystal structure of Mn_x_Zn_1−x_F nanoparticles requires X-ray diffraction (XRD) analysis in Fig. 2C. All of samples affirm a crystal unit with the spinel cubic structure for the various Mn_*x*_Zn_1–*x*_F nanoparticles (JCPDS card no. 22-1012) under 2θ values ranging from 20 to 70°. The diffraction peaks were contributed from the indexed crystal planes, (220), (311), (400), (422), (511), and (440), respectively. In addition, a progressive shift of (311) diffraction peak toward a lower angle with decrease of *x* (Fig. 2D), indicating an expansion of the crystal unit due to introduction of Zn^2+^ ion replace Mn^2+^ ion. According to Scherrer’s equation [30], the average crystallite sizes of the NPs doped with different *x* content are calculated: 9.73, 9.37, 9.77, 9.47, 9.03, and 9.73 nm, respectively. The results were almost consistent with the particle size statistics in Fig. 2B (PDI = 0.09, 0.11, 0.08, 0.08, 0.13, and 0.15, respectively). The magnetic heating efficacy of Mn_x_Zn_1−x_F particles is closely related to their magnetic properties [31].

**Fig. 3:**
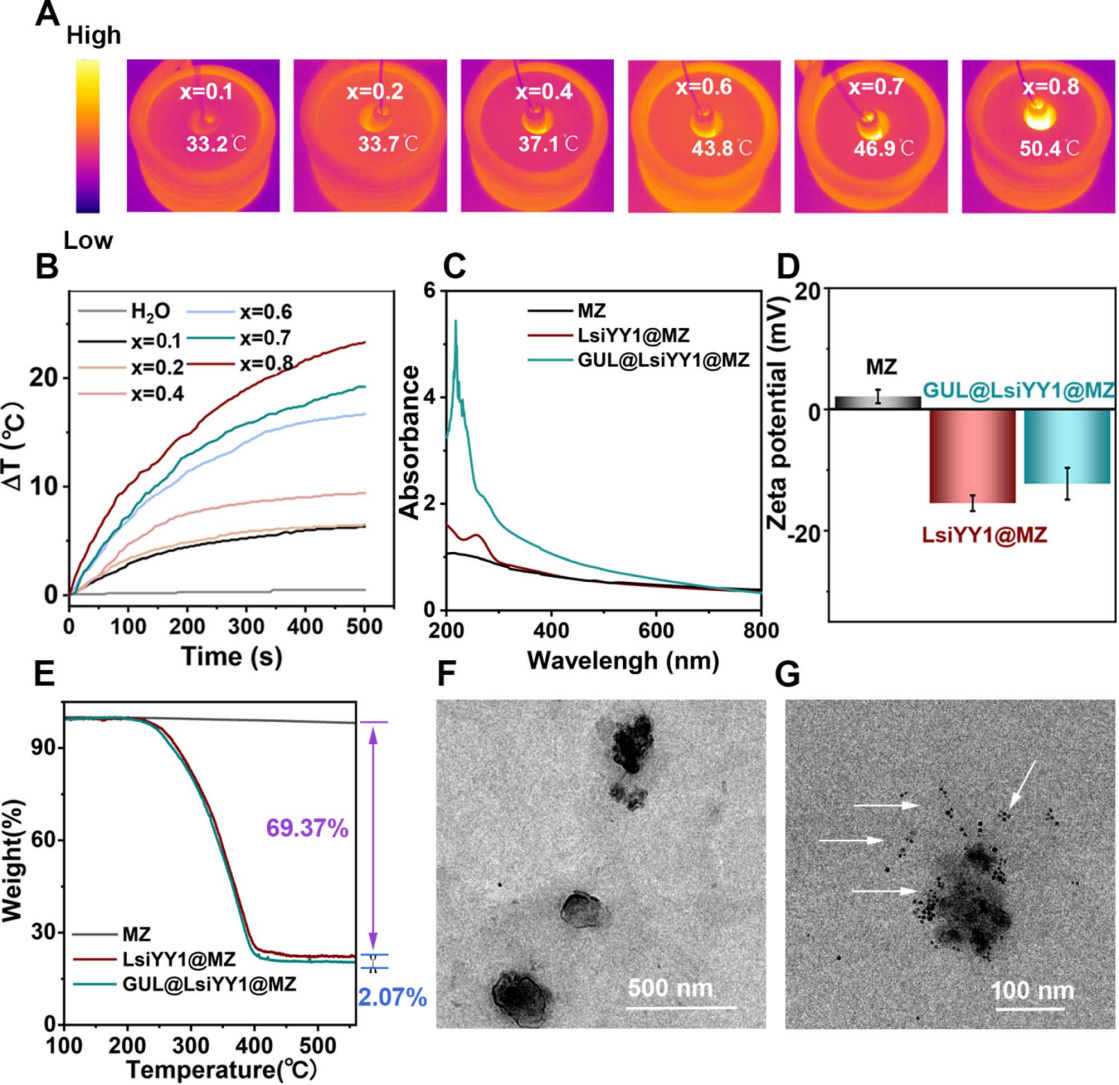
Magnetothermal performance of Mn_*x*_Zn_1–*x*_F at concentrations of 0.5 mg/ml under AMF for 500 s: (**a**) profile of thermal image. (**b**) Time-dependent temperature curves. Characterization of MZ, LsiYY1@MZ and GUL@LsiYY1@MZ nanohybrids: (**c**) UV-vis spectra of aqueous solution. (**d**) Zeta potentials. (**e**) TG curves. (**f-g**) TEM images of GUL@LsiYY1@ MZ nanohybrids treated without AMF and under AMF 15 min conditions (The white arrows denote the release of MZ nanoparticles resulting from the rupture of the liposome structure upon temperature elevation)

To evaluate the potential of Mn_x_Zn_1−x_F particles as magnetic heating agents, the field-dependent magnetization (M-H) curves were explored at 300 K (Fig. 2F and H). The saturation magnetization (σ_s_) values of Mn_x_Zn_1−x_F nanoparticles with different *x* content are 21.25, 29.23, 31.82, 37.31, 34.58 and 31.93 emu/g, respectively. The σ_s_ values showed a tendency to increase and then decrease upon further substitution. Similar behaviour has been reported σ_s_ can be maximized at *x* = 0.6 and explained as the competition between the A-B exchange and B-B exchange in the spinel ferrite [20]. Figure 2G revealed that the nanoparticles had almost no coercivity and remanent magnetization, indicating Mn_x_Zn_1−x_F nanoparticles had superparamagnetic behavior. This property ensured that Mn_x_Zn_1−x_F nanoparticles would do spontaneously aggregate, which were suitable for biomedical application. Combined with the above analysis, Mn_0.6_Zn_0.4_Fe_2_O_4_ processed the highest σ_s_ and no significant hysteresis. High magnetic magnetothermal performances of nanoparticles were crucial for application in the biological field. The temperatures of Mn_x_Zn_1−x_F nanoparticle solutions (0.5 mg/mL) were monitored by thermal imaging camera under AMF (324 Oe, 270 kHz) for 500 s as shown in Fig. 3A. It was difficult to reach the temperature to reach magnetothermal therapy, when *x* is less than 0.6. Heating curves of Mn_x_Zn_1−x_F nanoparticle solutions (Fig. 3B) were observed that the temperature increases significantly with increasing of *x*. Moreover, the rate of temperature increased of *x* = 1 sample was faster than other samples. When *x* = 0.6, the temperature reached approximately 43.8 ◦C (*SAR* = 910 W/g), showcasing its superior magnetothermal properties in MHT. Hence, Mn_0.6_Zn_0.4_Fe_2_O_4_ have the best superparamagnetism in biomedical application for subsequent experimental exploration.

### Characterization and property of GUL@LsiYY1@MZ nanohybrids

To improve targeting performance for prostate cancer and avoid the rapid clearance of nanoparticles from the body, MZ and siRNA were encapsulated within thermosensitive liposomes. Additionally, the small molecule GUL, which targets PSMA, was conjugated to the exterior, resulting in the formation of efficient, spherical nanoparticles designated as GUL@LsiYY1@MZ (Fig. 3E). The particle sizes were further confirmed by DLS analysis which had an average hydrodynamic diameter of 250 nm with a PDI of 0.339 (Figure S2A). The slightly larger sizes compared to TEM results is consistent with DLS measuring the hydrodynamic diameter. This discrepancy was commonly attributed to the fact that DLS measured the hydrodynamic diameter, including the hydration layer surrounding the nanoparticles. To validate the successful construction of the nanohybrids, the UV-vis absorption spectra, zeta potential, and TGA of the MZ, LsiYY1@MZ, and GUL@LsiYY1@MZ nanohybrids in aqueous solution were measured as depicted in Fig. 3C-E. As shown in Fig. 3C, a distinct absorption peak at 260 nm emerged, indicating the successful integration of siYY1, potentially containing nucleic acid components, as evidenced by the characteristic absorbance of nucleic acids at this wavelength [32]. Additionally, the introduction of GUL into GUL@LsiYY1@MZ resulted in the appearance of a characteristic absorption peak at 290 nm, corresponding to the GUL molecule [33]. These results collectively confirmed the hierarchical GUL@LsiYY1@MZ: the liposome coating enhanced colloidal stability, siYY1 introduced nucleic acid-related absorption features, and GUL introduced surface-targeting functionalities. Subsequently, the zeta potential of samples evaluated in Fig. 3D, the zeta potential value of MZ and LsiYY1@MZ are 4.3 and − 15.4 mV, respectively. The positive zeta potential made the MZ nanoparticles in favor of connection with negatively charged liposomes. Upon further modification with GUL, the zeta potential of GUL@LsiYY1@MZ showed a negative charge of -12.2 mV, possibly due to the presence of GUL increasing the negative charge on the surface. These results further confirmed the successful fabrication and surface functionalization of GUL@LsiYY1@MZ nanohybrids. To further confirm the successful construction of the nanohybrids, TGA was performed in the Fig. 3E, showing the weight loss profiles as a function of temperature. MZ exhibits minimal wight loss (∼ 2.07%), reflecting high thermal stability. In contrast, both LsiYY1@MZ and GUL@LsiYY1@MZ displayed significant weight loss beginning around 300 °C, with an overall mass loss of approximately 69.37% for GUL@LsiYY1@MZ. This enhanced degradation is attributed to the presence of organic components (liposomes, siYY1, and GUL), thereby further validating the successful hybrid construction.

Magnetic properties of GUL@LsiYY1@MZ were also evaluated (Figure S2B), showing σ_s_ of 29 emu/g with negligible coercivity and remanence, highlighting its superparamagnetic behavior, which was advantageous for MHT. Additionally, GUL@LsiYY1@MZ could rapidly aggregate under an external magnetic field, as demonstrated in Figure S2D. Stability is crucial for the practical biomedical application of nanoparticles. The colloidal stability of GUL@LsiYY1@MZ was evaluated under different physiological conditions, including deionized water, PBS, normal saline, and complete cell culture medium at room temperature (Figure S3). The GUL@LsiYY1@MZ nanohybrids exhibited excellent stability in all tested media. Considering the pH difference between the tumor microenvironment and normal tissues, we further investigated the stability of GUL@LsiYY1@MZ at various pH values (Figure S4). GUL@LsiYY1@MZ nanohybrids remained stable across a pH range of 5.5 to 7.5, further supporting their potential for in vivo therapeutic applications.

To evaluate the temperature-responsive release capabilities of GUL@LsiYY1@MZ, the hybrids were exposed to AMF. As shown in Fig. 3G, the nanocluster structure of GUL@LsiYY1@MZ degraded and break apart into numerous MZ nanocrystals (white arrows) after treatment. This structural breakdown confirmed the thermosensitive liposome under elevated temperatures. To systematically evaluate the temperature-responsive GUL@LsiYY1@MZ release of siYY1, we conducted comprehensive release studies under varying thermal conditions and GUL@LsiYY1@MZ concentrations. The temperature was precisely controlled at AMF exposure durations, with GUL@LsiYY1@MZ tested across a concentration gradient as depicted in Figure S5. Our studies identified 100 µg/mL as the optimal concentration, at which the system achieved the target hyperthermia temperature of 42 °C within 500 s of AMF exposure. The efficient encapsulation of siRNA within GUL@LsiYY1@MZ enables investigation of its stimuli-responsive release behavior under different thermal conditions (36–42 °C). Quantitative analysis revealed temperature-dependent release profiles, with minimal leakage (< 5%) at physiological temperature (36 °C) but significant payload liberation at hyperthermic conditions (Figure S6). Notably, the system exhibited initial siRNA release at 42 °C (86.3% after 30 min), reaching highest cumulative release. The 42 °C was selected for subsequent biological experiments as it represents a clinically achievable hyperthermia temperature while still maintaining substantial siRNA release. This thermosensitive behavior, mediated by temperature-dependent liposome disruption, significantly enhances gene delivery efficiency during magnetic hyperthermia. The dual responsiveness of GUL@LsiYY1@MZ makes it particularly promising for combined MHT and gene therapy against prostate cancer, making GUL@LsiYY1@MZ highly promising for combined MHT and gene therapy against prostate cancer.

### In vitro cell uptake and cytotoxicity assays

**Fig. 4:**
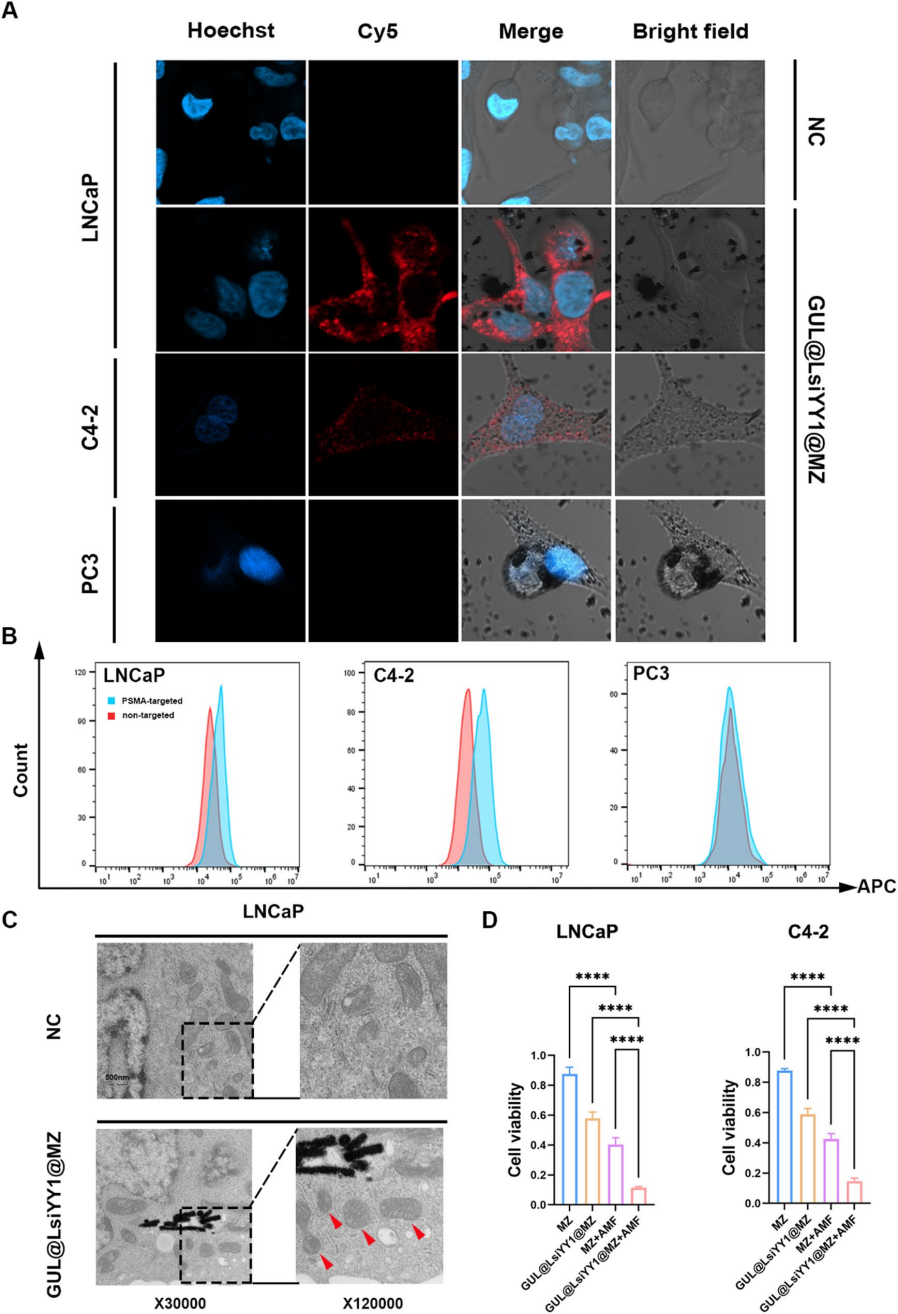
Cell viability of PCa cell lines treated with different nanoparticles. (**a**) Confocal images showing cell uptake of Cy5-labeled GUL@LsiYY1@MZ nanoparticles in LNCaP, C4-2 and PC3 cells. (**b**) Flow cytometry was used to analyze the cellular uptake efficiency of PSMA-targeted and non-targeted nanoparticles in LNCaP, C4-2 and PC3 cells. (**c**) Transmission electron microscopy demonstrated the endocytosis of GUL@LsiYY1@MZ nanoparticles by LNCaP cells. (**d**) Viability of LNCaP and C4-2 cells under different treatments at a concentration of 100 µg/mL for 48 h by CCK-8 assays (*n* = 5). One-way ANOVA with Tukey’s post-hoc test was used for multi-group comparisons. Significance levels: *p < 0.05, **p < 0.01, ***p < 0.001, ****p < 0.0001

After 4 h of incubation, the Cy5-labeled GUL@LsiYY1@MZ nanomaterials were effectively internalized into PSMA positive LNCaP and C4-2 cells, as demonstrated in Fig. 4A. However, we did not observe a significant fluorescence signal in PSMA-negative PC3 cells. In addition, to determine PSMA-targeting efficiency, we performed flow cytometry in the PSMA-targeted and non-targeted groups (Fig. 4B). The results showed that the PSMA-targeted group had a 2-to 3-fold increase in MFI compared to the non-targeted group in LNCaP and C4-2 cells, but not in PC3 cells (Figure S7). This indicates that GUL@LsiYY1@MZ has excellent targeting ability and can only be recognized, internalized by PSMA-expressing cells. Transmission electron microscopy (TEM) further confirmed the endocytosis of these nanoparticles into LNCaP cells. Notably, ferroptosis was observed in the mitochondria, characterized by a reduction in mitochondrial volume, an increase in mitochondrial membrane density, and a decrease in mitochondrial cristae, as illustrated in Fig. 4C. To assess both the cytotoxicity and anti-tumor efficacy of different treatment, a cell viability assay was conducted (Fig. 4D). The results demonstrated that the cell viability rates for the GUL@LsiYY1@MZ + AMF group, the MZ + AMF group, the GUL@LsiYY1@MZ group, and the MZ group were 11%, 40%, 58%, and 87%, respectively. These findings suggest that the GUL@LsiYY1@MZ + AMF formulation exhibited superior anti-tumor efficacy compared to both the MZ + AMF and GUL@LsiYY1@MZ formulations alone, while maintaining low toxicity at a concentration of 100 µg/mL. In conclusion, the GUL@LsiYY1@MZ formulation demonstrated enhanced anti-tumor effects through the release of siYY1 in conjunction with AMF treatment, and the nanoparticles were found to be biocompatible.

### GUL@LsiYY1@MZ under AMF trigger ferroptosis in prostate cancer therapy

**Fig. 5:**
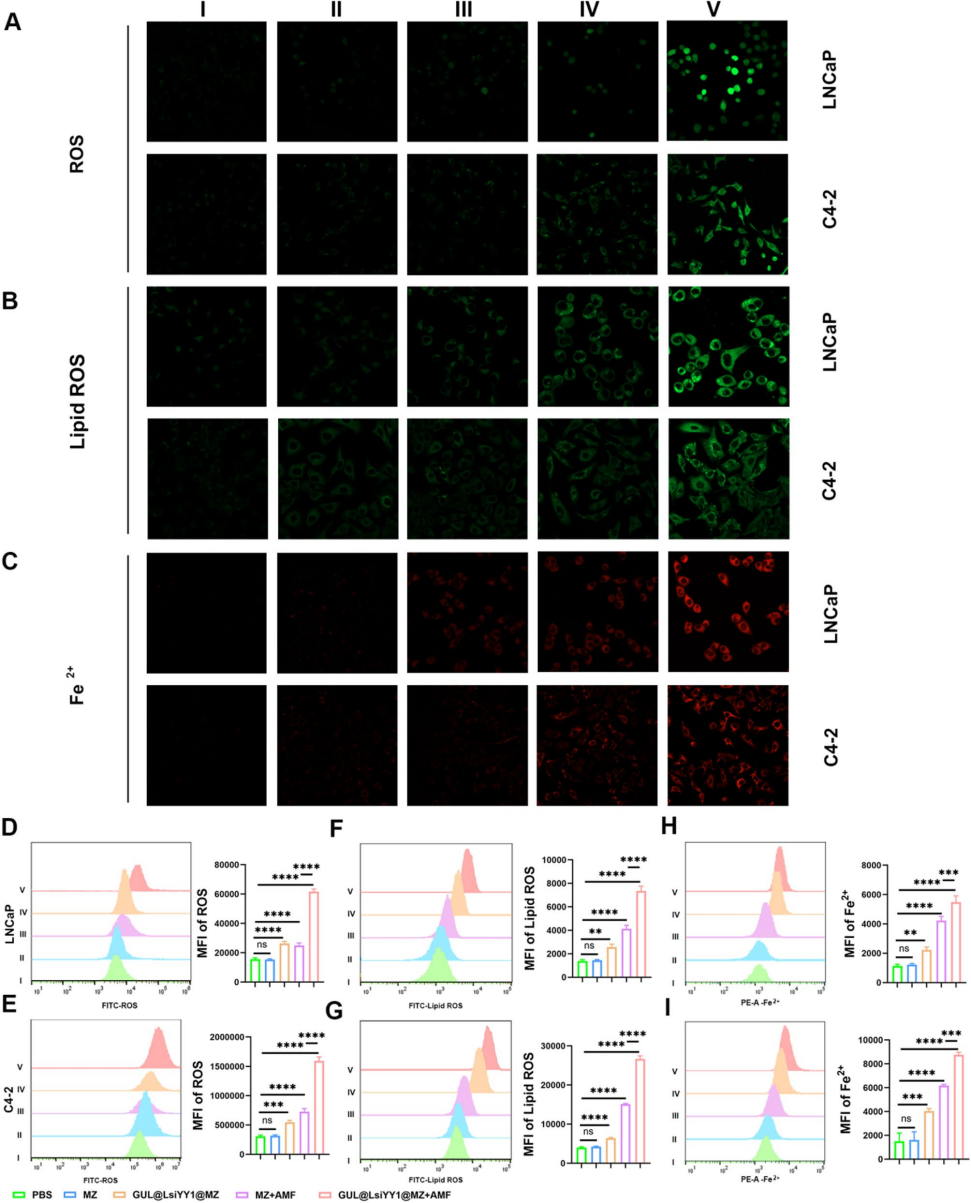
GUL@LsiYY1@MZ Nanomaterials combined with AMF trigger ferroptosis in prostate cancer therapy. CLSM images of LNCaP and C4-2 cells stained with (**a**) DCFH-DA, (**b**) C11-BODIPY and (**c**) FerroOrange in different groups. Flow cytometry analyses of intracellular (**d**, **e**) ROS, (**f**, **g**) LPO and (**h**, **i**) Fe ^2+^ generation in LNCaP and C4-2 cells; I, NC; II, MZ; III, GUL@LsiYY1@MZ; IV, MZ + AMF; V, GUL@LsiYY1@MZ + AMF. Data are reported as mean values ± SD. One-way ANOVA with Tukey’s post-hoc test was used for multi-group comparisons (*n* = 3). Significance levels: n.s, not significant; *p < 0.05; **p < 0.01; ***p < 0.001; ****p < 0.0001

In the subsequent phase of our study, we sought to ascertain whether GUL@LsiYY1@MZ could induce ferroptosis in the context of prostate cancer therapy. The endoperoxide linkages are capable of reacting with ferrous ions, resulting in the accumulation of substantial amounts of reactive oxygen species (ROS) through a Fenton-like reaction, thereby causing oxidative damage to cells. The production of ROS in PCa cells was subsequently assessed using DCFH-DA. In comparison to the negative control, PCa cells treated with MZ exhibited only minimal fluorescence intensity (Fig. 5A). In contrast, the MZ + AMF groups demonstrated increased fluorescence intensity, which was further amplified following treatment with GUL@LsiYY1@MZ + AMF. This suggests that AMF treatment facilitates the release of siRNA via a thermosensitive mechanism, synergistically producing a maximal amount of ROS in PCa cells due to the pronounced oxidative damage induced by the combined effects of GUL@LsiYY1@MZ nanoparticles and AMF treatment. To further substantiate ROS production, qualitative analysis was conducted using flow cytometry. The results indicated that, relative to the MZ + AMF group, the GUL@LsiYY1@MZ + AMF group exhibited a more pronounced accumulation of ROS (Figs. 5D, E), suggesting that AMF treatment enhances the oxidative damage associated with YY1 silencing in PCa cells.

Upon excessive accumulation of ROS within cells, a portion is converted into lipid peroxidation (LPO), a critical marker of ferroptosis [34]. Consequently, we employed C11-BODIPY to assess the intracellular levels of lipid peroxidation. As depicted in Fig. 5B, minimal fluorescence was detected in the MZ and control groups, indicating negligible LPO production. Conversely, the MZ + AMF group exhibited a marked increase in green fluorescence, suggesting the generation of a moderate amount of LPO. Notably, following incubation with GUL@LsiYY1@MZ under AMF treatment, a pronounced fluorescence was observed. This finding elucidates that the combined effect of GUL@LsiYY1@MZ and AMF treatment facilitated substantial LPO production, which is posited to contribute to its heightened cytotoxicity compared to AMF treatment alone. Furthermore, flow cytometry results corroborated the CLSM findings, indicating maximal LPO production in the GUL@LsiYY1@MZ NPs + AMF group within PCa cells (Fig. 5F, G). Therefore, this nanoplatform effectively disrupts the cellular redox balance in PCa cells, inducing ferroptosis through the accumulation of intracellular LPO.

Intracellular ferrous ion levels were evaluated using FerroOrange. As illustrated in Fig. 5C, treatment with MZ and GUL@LsiYY1@MZ resulted in a slight increase in fluorescence intensity in PCa cells compared to the control group. Notably, the MZ + AMF and GUL@LsiYY1@MZ + AMF groups exhibited the most pronounced fluorescence, aligning with the flow cytometry results depicted in Fig. 5H and I. This observation may be attributed to the enhanced release of ferrous ions from the nanomaterials through a thermally sensitive mechanism under AMF treatment, directly inducing ferroptosis via the Fenton reaction [35]. Additionally, siRNA decreased YY1 protein production in the cytoplasm, limiting its nuclear entry and binding to promoter regions of target genes, thereby inhibiting transcription of oxidative stress-related genes and disrupting cellular REDOX balance (Fig. 7H).

Furthermore, to further explore whether the observed reduction in cell viability was mediated through ferroptosis, cells were treated with the ferroptosis inhibitor Fer-1 and the iron chelator DFO to assess the recovery of cell viability. The results demonstrated that Fer-1 was able to partially restore cell viability in the GUL@LsiYY1@MZ + AMF, MZ + AMF, and GUL@LsiYY1@MZ groups to varying extents. DFO restored cell viability in the GUL@LsiYY1@MZ + AMF and MZ + AMF groups. However, cell viability in the MZ group could not be restored by either Fer-1 or DFO (Figure S8A-B). These findings confirm that AMF-triggered oxidative cascades are responsible for executing ferroptosis. Notably, GUL@LsiYY1@MZ, in the absence of AMF, also induces ferroptosis through the transient release of siYY1, which mediates the suppression of SLC7A11, thereby collapsing glutathione-dependent antioxidant defenses independently of iron overload (Figure 7H). In contrast, MZ alone (without AMF) resulted in only a mild reduction in cell viability (15 to 20%), primarily through non-oxidative mechanisms.

### Oxidized lipidomics analyses

**Fig. 6:**
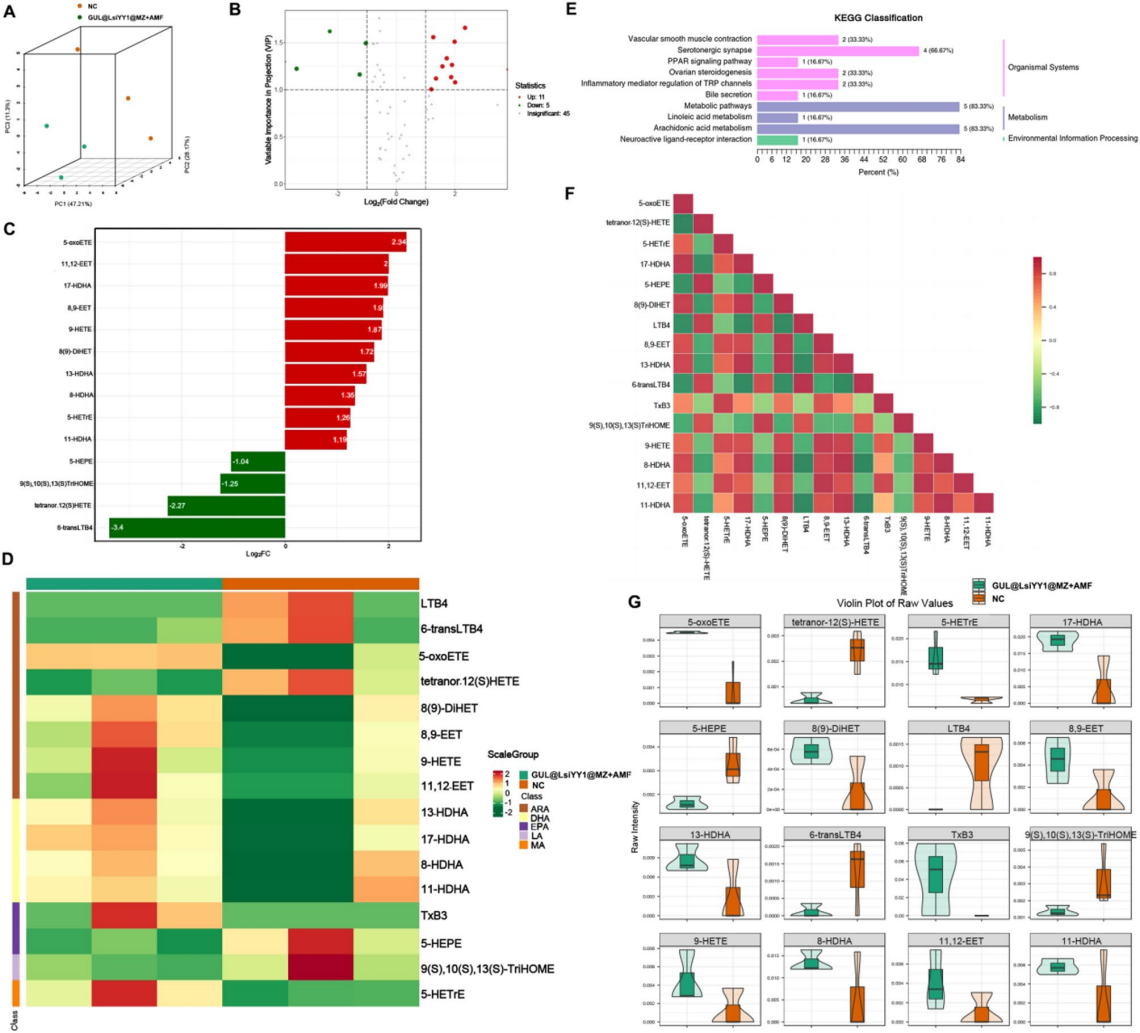
Oxidized lipidomics analyses. (**a**) 3D-PCA of the control and GUL@LsiYY1@MZ + AMF groups. (**b**) Volcano plot of the control and GUL@LsiYY1@MZ + AMF groups. (**c**) Bar graph of differentially regulated oxidized lipid products. (**d**) Heatmap of differentially regulated oxidized lipid products the control and GUL@LsiYY1@MZ + AMF groups. (**e**) KEGG enrichment pathway analysis of differentially regulated oxidized lipid products. (**f**) Pearson correlation analysis of differentially regulated oxidized lipid products. (**g**) Volin plot of each raw value

Ferroptosis is a cell death mechanism characterized by iron-dependent peroxidation of phospholipids (PL) [36]. Conceptually, ferroptosis can be considered a consequence of cellular metabolism, wherein oxygen and iron, as essential components of metabolic processes, lead to the unavoidable generation of ROS. When a specific type of ROS, phospholipid peroxides (PLOOH), is not adequately neutralized and accumulates to a level that compromises plasma membrane integrity, cells undergo ferroptosis. To investigate this process, we conducted a lipidomic analysis of oxidized lipid metabolites in the GUL@LsiYY1@MZ + AMF group compared to the NC group. Principal component analysis (PCA) was employed to assess the degree of sample aggregation and dispersion (Fig. 6A). The PCA results indicated that the oxidized lipid metabolites in the GUL@LsiYY1@MZ + AMF group exhibited significant differences from those in the NC group, with distinct aggregation patterns. Further differential metabolite analysis between the two groups identified 11 significantly up-regulated and 5 significantly down-regulated oxidized lipid metabolites (Fig. 6B, C). The study identified the presence of eight arachidonic acids (AA), four docosahexaenoic acids (DHA), two eicosapentaenoic acids (EPA), one eicosanotrienoic acid (MA), and one linoleic acid (LA), all of which are classified as polyunsaturated fatty acids (PUFAs) (Fig. 6D). Analysis using the KEGG pathway revealed that these compounds were predominantly enriched within metabolic pathways (Fig. 6E). Within cellular environments, the substrates for PL peroxidation are phospholipids containing PUFA chains at the sn2 position. In the presence of biologically active iron, PUFA-containing phospholipids (PUFA-PLs) can undergo conversion to PLOOH through both enzymatic and non-enzymatic lipid peroxidation processes [37]. In this study, treatment of PCa cells with GUL@LsiYY1@MZ + AMF resulted in the production of a substantial quantity of PUFAs, surpassing the cells’ intrinsic protective mechanisms and ultimately inducing ferroptosis. However, Pearson correlation analysis indicated that some PUFAs exhibited negative correlations, implying that the metabolic alterations induced by GUL@LsiYY1@MZ + AMF treatment may be concentrated within a specific pathway (Fig. 6F). Highly unsaturated phosphatidylethanolamines and phosphatidylcholines, which serve as signaling molecules, are particularly susceptible to oxidation, thereby triggering ferroptosis in cells, especially in the presence of arachidonic acid [38]. Recent studies have reported that other PUFAs, such as DHA, EPA, and LA, can induce ferroptosis in cells [39]. In this research, it was demonstrated that several polyunsaturated forms of arachidonic acids (AAs) (5-oxoETE, 8(9)-DiHET, 8,9-EET, 9-HETE, and 11,12-EET), DHAs (13-HDHA, 17-HDHA, 8-HDHA, and 11-HDHA), and EPA (TxB3) were significantly upregulated following GUL@LsiYY1@MZ + AMF treatment (Fig. 6F, G). In summary, the oxidized lipidomics analyses revealed that the increase in PUFAs and the accumulation of LPO were implicated in ferroptosis induced by GUL@LsiYY1@MZ + AMF treatment in PCa cells.

### Transcriptomic analyses

**Fig. 7:**
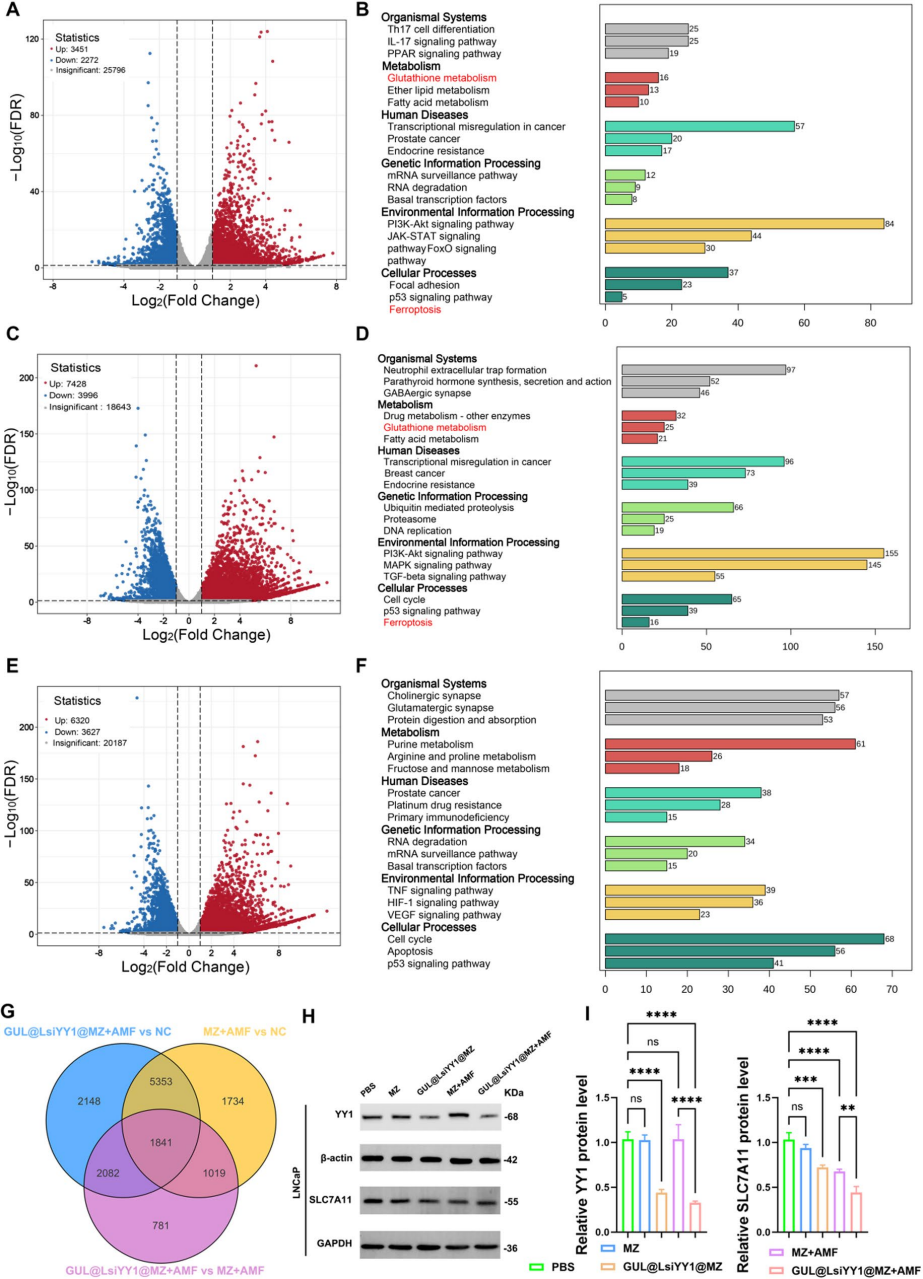
Transcriptomic analyses of PCa cells after different treatments. (**a**) Volcano diagram of GUL@LsiYY1@MZ + AMF v.s NC, the cutoff value is set to | log_2_ (fold change) | ≥ 1 and p.adj ≤ 0.05. (**b**) KEGG enrichment analyses of DEGs in GUL@LsiYY1@MZ + AMF v.s NC. (**c**) Volcano diagram of GUL@LsiYY1@MZ + AMF v.s MZ + AMF, the cutoff value is set to | log_2_ (fold change) | ≥ 1 and p.adj ≤ 0.05. (**d**) KEGG enrichment analyses of DEGs in GUL@LsiYY1@MZ + AMF v.s MZ + AMF. (**e**) Volcano diagram of MZ + AMF v.s NC groups, the cutoff value is set to | log_2_ (fold change) | ≥ 1 and p.adj ≤ 0.05. (**f**) KEGG enrichment analyses of DEGs in MZ + AMF v.s NC. (**g**) Venn diagram of the three groups of DEGs. (**h**) Western blotting assays showed the protein level of YY1 and SLC7A11 in LNCaP cell after different treatments. (**i**) Relative protein levels of YY1 and SLC7A11 under different treatments. Data are reported as mean values ± SD. One-way ANOVA with Tukey’s post-hoc test was used for multi-group comparisons (*n* = 3). Significance levels: n.s, not significant; *p < 0.05; **p < 0.01; ***p < 0.001; ****p < 0.0001

We analyzed the effects of various nanoparticles on gene expression in LNCaP cells using RNA sequencing and pairwise comparisons. Volcano plots (Fig. 7A and C, and E) show differentially expressed genes (DEGs) between GUL@LsiYY1@MZ + AMF and NC, GUL@LsiYY1@MZ + AMF and MZ + AMF, and MZ + AMF and NC. The analysis identified significant changes in mRNA expression, with thousands of genes up- or down-regulated in each comparison, indicating notable shifts in gene expression patterns due to nanoparticle treatments.

Glutathione (GSH) and GPX4 play a pivotal role in the antioxidant defense system. The impairment of this intracellular antioxidant mechanism, attributable to various factors, leads to the accumulation of lipid peroxides, which ultimately induces ferroptosis [40]. KEGG pathway enrichment analysis demonstrated that the DEGs across the three groups were significantly linked to ferroptosis pathways, particularly those involving GSH metabolism (Fig. 7B and D, and F). This indicates that the combined treatment of GUL@LsiYY1@MZ with AMF disrupts intracellular redox homeostasis, resulting in lipid peroxide accumulation and culminating in ferroptosis.

Subsequently, we determined the intersection of DEGs across the three groups, culminating in the identification of 1,841 genes (Fig. 7G). To explore the potential role of these novel DEGs in the regulation of cellular antioxidant mechanisms, we conducted a cross-referencing analysis with genes associated with ferroptosis. This analysis identified SLC7A11 as a significantly differentially expressed gene. Cysteine functions as the rate-limiting precursor for GSH synthesis, with intracellular cysteine primarily supplied via SLC7A11-mediated cystine uptake. Corroborating this, prior studies have shown that the depletion of cystine from cell culture media or the inactivation of SLC7A11 through genetic ablation or pharmacological inhibition triggers ferroptosis in various cancer cell lines. Conversely, the overexpression of SLC7A11 in cancer cells enhances GSH biosynthesis and imparts resistance to ferroptosis [41]. Consequently, we conducted western blotting analyses to determine the knockdown efficiency of YY1 under different treatment conditions and further explore whether various treatments could influence intracellular cystine transport by inhibiting SLC7A11, ultimately resulting in reduced GSH synthesis. The results showed that GUL@LsiYY1@MZ + AMF significantly reduced the protein level of YY1 and SLC7A11(Fig. 7H-I, Figure S9).

### In vivo antitumor efficacy

**Fig. 8:**
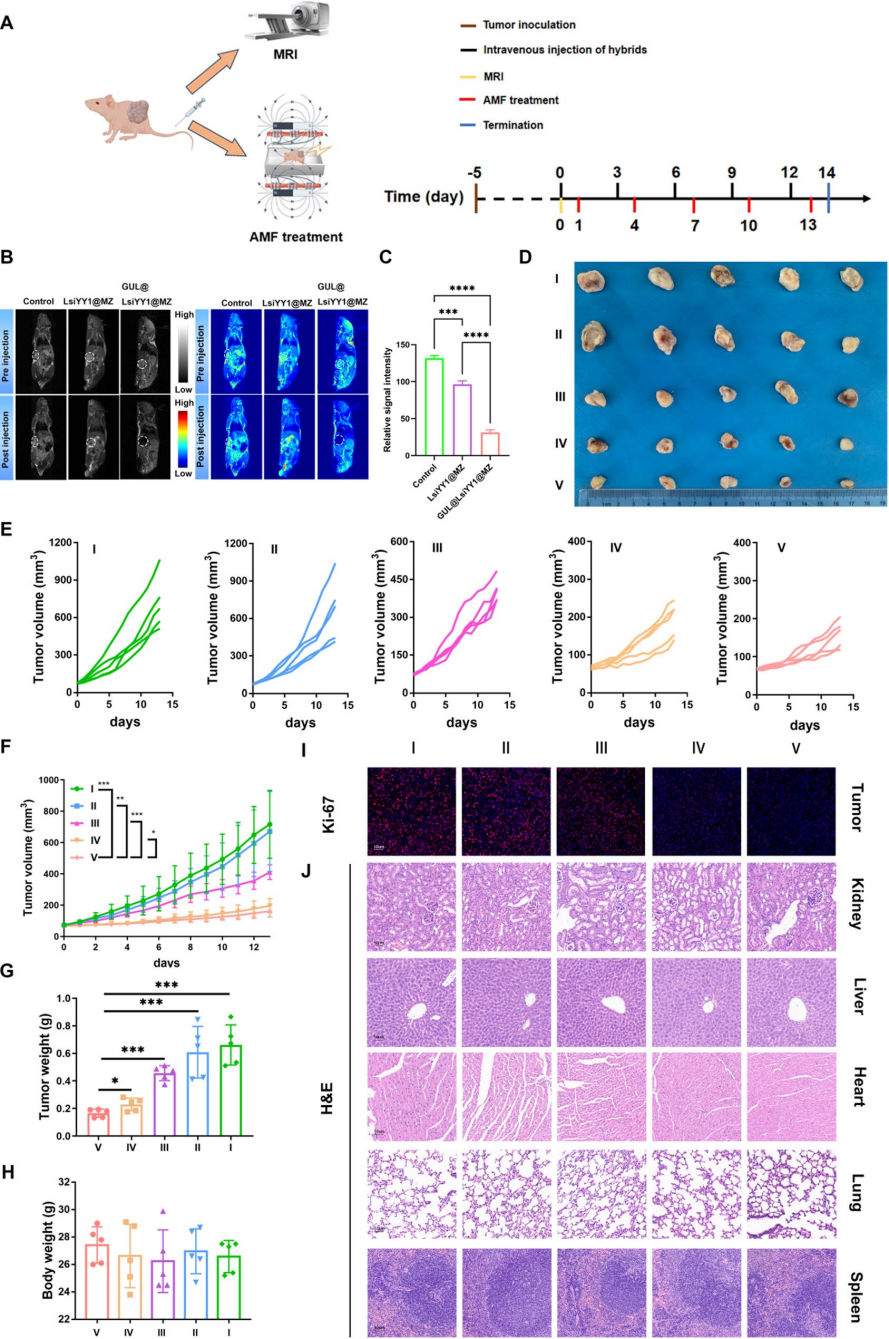
In vivo antitumor efficacy. (**a**) Scheme of cancer therapy and MRI diagnosis in PCa tumor models. (**b**) T_2_-weighted MRI images of mice treated with PBS, LsiYY1@MZ, and GUL@LsiYY1@MZ before and after injection. The corresponding pseudocolor images were presented to enhance visual contrast. (**c**) Quantitative bar graph of MR Signal, *n* = 3. (**d**) Photographs of tumors after therapy. (**e-h**) The curves of tumor volume, *n* = 5 (**f**); tumor weight, *n* = 5 (**g**); body weight changes during the 14-day treatment period, *n* = 5 (**h**). (**i**) Immunostaining images of Ki-67 in tumor tissues after different treatment. (**j**) H&E staining images of organs dissected from each group on the 14th day. I, PBS; II, MZ; III, GUL@LsiYY1@MZ; IV, MZ + AMF; V, GUL@LsiYY1@MZ + AMF. Data are reported as mean values ± SD. One-way ANOVA with Tukey’s post-hoc test was used for multi-group comparisons. Significance levels: n.s, not significant; *p < 0.05; **p < 0.01; ***p < 0.001; ****p < 0.0001

The in vivo anti-tumor efficacy and MRI characteristics of GUL@LsiYY1@MZ nanohybrids were evaluated using the LNCaP subcutaneous tumor model (Fig. 8A). In vitro MRI tests were tested using GUL@LsiYY1@MZ with varying Fe concentrations to assess the T_2_ contrast performance (Figure S10). The results demonstrated a strong Fe concentration-dependent signal reduction in T_2_-weighted images, accompanied by a progressive increase in T_2_ relaxivity (Figure S10B), with the maximum r_2_ value reaching 206 mM^− 1^·s^− 1^ (Figure S10D). Based on the in vitro relaxivity measurements, the calculated r_2_/r_1_ ratio was 98 (Figure S10C) which exceeded 10 [42], confirming that the GUL@LsiYY1@MZ behave as efficient T_2_-weighted MRI contrast agents. Based on these findings, we conducted in vivo T_2_-weighted MRI after intravenous injection to evaluate the imaging performance in the LNCaP tumor model, as below groups: control (pre injection), LsiYY1@MZ (post injection, non-targeted) and GUL@LsiYY1@MZ (post injection, targeted) groups. T_2_-weighted MR imaging of prostate tumor cells (Fig. 8B) revealed that noticeable darkening of the T_2_-weighted MRI images was observed at 24 h [43, 44], suggesting preferential accumulation of the nanohybrids in the tumor region (Fig. 8B). Quantitative analysis (Fig. 8C) confirmed post-injection signal intensities of 131% (control), 96% (LsiYY1@MZ), and 30% (GUL@LsiYY1@MZ) relative to the pre-injection baseline. Crucially, the targeted GUL@LsiYY1@MZ group exhibited a significantly greater signal decrease compared to both the control and non-targeted groups. These results quantitatively demonstrate the enhanced accumulation of GUL@LsiYY1@MZ in tumor tissue via active targeting, leading to stronger T_2_ contrast effects. GUL@LsiYY1@MZ enables real-time, MR-guided visual assessment of treatment delivery, providing a critical tool for monitoring targeted treatment efficacy.

To assess the anti-tumor effects, LNCaP tumor-bearing male nude mice were randomly assigned to five groups (*n* = 5). Intravenous injections of the nanohybrids were administered every three days, followed by AMF treatment the subsequent day. Results indicated that both MZ and GUL@LsiYY1@MZ exhibited limited tumor inhibition (Figs. 8D-G). However, the combination of GUL@LsiYY1@MZ with AMF treatment significantly impeded tumor growth in vivo. This suggests that GUL@LsiYY1@MZ nanoparticles not only achieve effective tumor site enrichment but also demonstrate enhanced anti-tumor efficacy when combined with AMF therapy. It is noteworthy that all experimental groups exhibited non-significant weight loss, indicating that the nanoparticles exerted minimal side effects on the mice (Fig. 8H). Furthermore, subcutaneous tumor tissues from each group were analyzed using the Ki-67 immunofluorescence assay to assess intratumoral cell proliferation activity. Consistent with the aforementioned findings, the GUL@LsiYY1@MZ + AMF group demonstrated the lowest Ki-67 fluorescence intensity, suggesting a significant inhibition of tumor growth in this group (Fig. 8I). To further evaluate the systemic toxicity of the nanoparticles, all mice were euthanized on the 14th day of treatment, and major organs (heart, liver, kidney, lung, and spleen) were harvested and subjected to H&E staining. The absence of notable tissue damage and pathological changes across the five groups indicates that the nanoparticles possess biological safety and hold promise as an effective nanoplatform for prostate cancer treatment (Fig. 8J).

## Study limitations

Our study investigated the GUL@LsiYY1@MZ nanoplatform that uses MHT to induce ferroptosis in PCa via YY1 silencing and SLC7A11 disruption. While preclinical models show effective tumor-specific siRNA delivery and ferroptosis activation, clinical translation is challenged by PCa heterogeneity. Key variables include interpatient differences in genetic/epigenetic profiles, PSMA expression, YY1 dependency, and redox homeostasis that impact efficacy. Current models cannot replicate this complexity. Bridging this gap requires systematic evaluation in clinically relevant systems, personalized strategies targeting heterogeneous biomarkers, and combinatorial approaches incorporating patient-specific biology for safe clinical implementation.

## Conclusion

The GUL@LsiYY1@MZ platform targets PSMA-positive prostate cancer cells and exhibits cascaded magnetoresponsive activity, facilitating magnetothermal-triggered siYY1 release and cellular entry. This platform integrates MRI-guided tumor localization with ferroptosis induction, achieved through SLC7A11 downregulation, which reduces glutathione synthesis, amplifying lipid peroxidation and iron-dependent cell death. Demonstrated in vivo efficacy in tumor suppression with minimal toxicity supports the potential development of integrated theranostic strategies for prostate cancer monitoring and treatment.

## Supplementary Information

Below is the link to the electronic supplementary material.

Supplementary Material 1

## Data Availability

On reasonable request, the corresponding author will provide raw data analyzed in this study.
