# Supplementary material for: PSMA-targeted theranostic nanoplatform achieves spatiotemporally precise therapy and triggers ferroptosis in prostate cancer treatment
Source: J Exp Clin Cancer Res. 2025 Sep 30;44:272. doi: 10.1186/s13046-025-03530-4 (PMC12487002; doi:10.1186/s13046-025-03530-4)
Supplement: Supplementary file 1 — Supplementary Material 1 [file 13046_2025_3530_MOESM1_ESM.doc]

**PSMA-targeted Theranostic nanoplatform achieves spatiotemporally precise therapy and Triggers Ferroptosis in prostate cancer treatment**

Linxue Zhang ^a^, Qi Sun ^a^, Dongxin Zheng ^b^, Xiang Huang ^b^, Zhong Yu ^a^, Zhongwen Lan ^a^, Wei Xiong ^b^*, Ke Sun ^a^*, Ruiji Liu ^b^*

^a^ *School of Materials and Energy, University of Electronic Science and Technology of China, Chengdu, 610054, P. R. China.*

^b^ *Department of Urology,* *Sichuan Provincial People's Hospital, School of Medicine,* *University of Electronic Science and Technology of China, Chengdu 610072, China.*

*** Correspondence**:

Corresponding author:

Ruiji Liu *, E-mail address: liuruiji66Dr@163.com;

Ke Sun *, E-mail address: ksun@uestc.edu.cn；

Wei Xiong*, E-mail address: 37033013@qq.com.


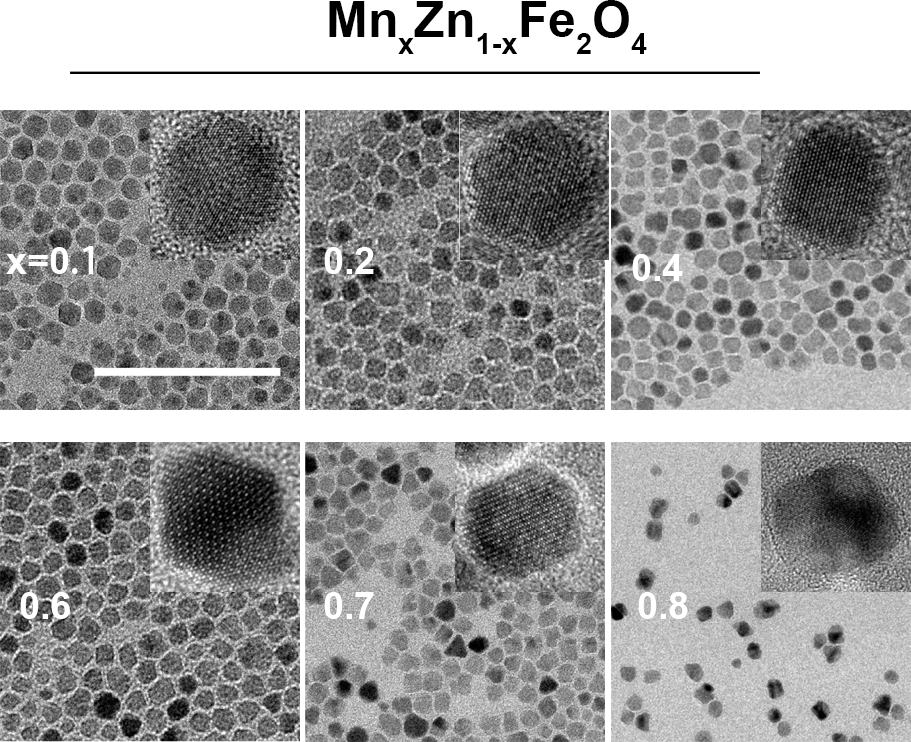


**Figure S1**. Morphology of nanoparticles with various Mn/Zn doping ratios Mn_x_Zn_1–x_F (x = 0.1, 0.2, 0.4, 0.6, 0.7, 0.8).


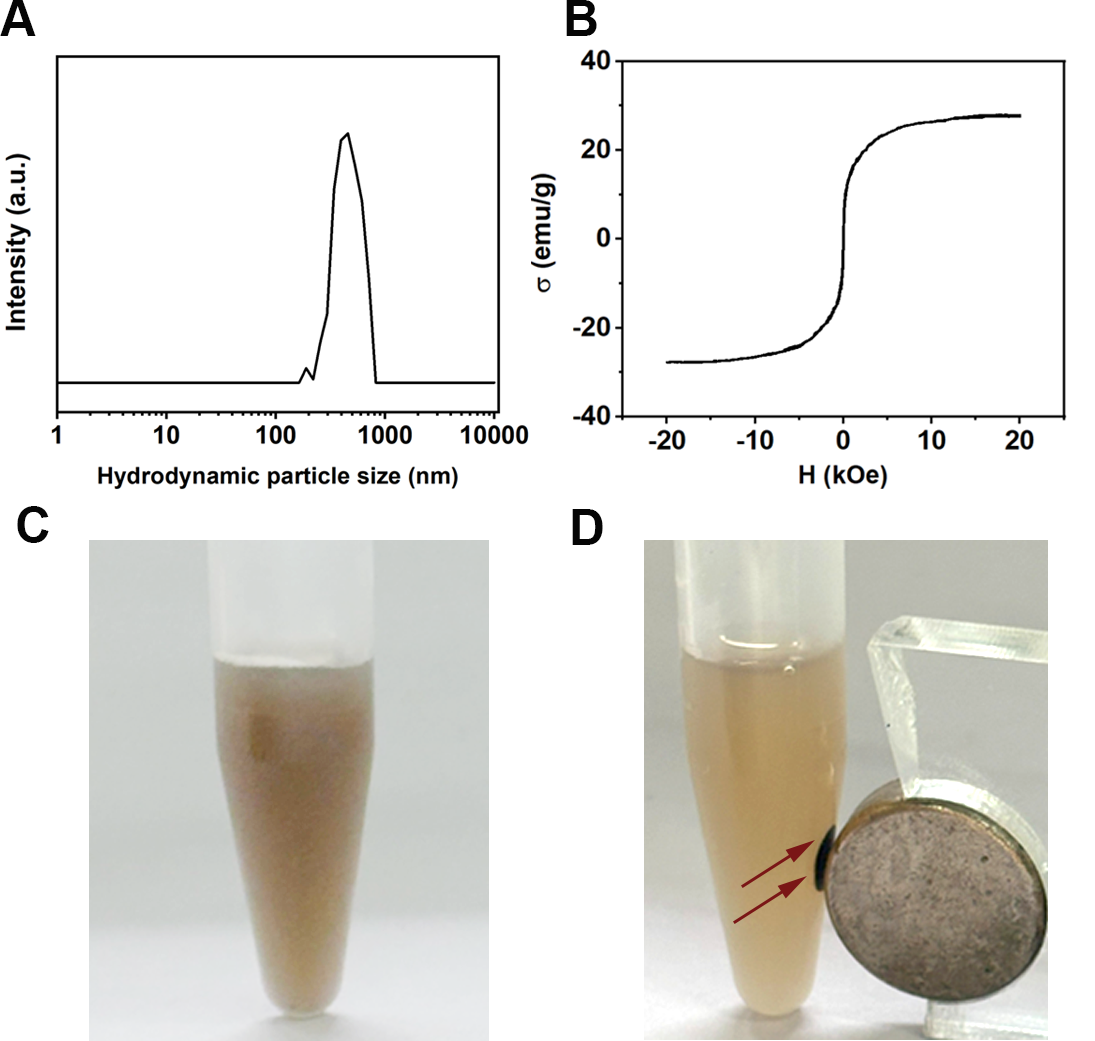


**Figure S2**. Characterization of the GUL@LsiYY1@MZ properties. **a** Hydrodynamic particle size distribution analysis, with PDI = 0.339. **b** Magnetic hysteresis curve. Photograph of the GUL@LsiYY1@MZ dispersion in water **c-d** with / without an external magnetic field.


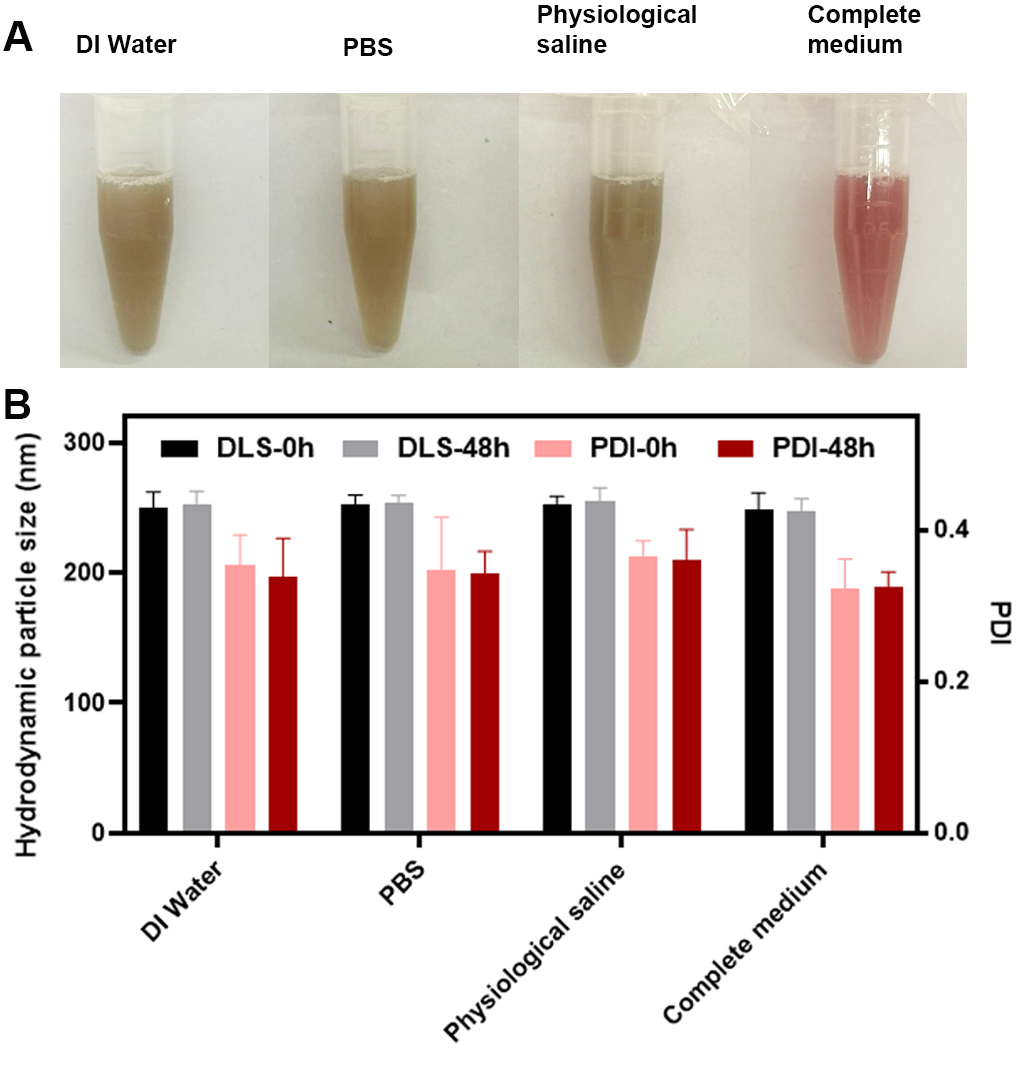


**Figure S3**. **a** Dispersion of GUL@LsiYY1@MZ in various solutions, including DI water, PBS, physiological saline solution and complete cell culture medium. **b** Stability of GUL@LsiYY1@MZ in different solutions monitored by DLS and PDI at 0 and 48 hours. Data are presented as mean ± SD (n=3).


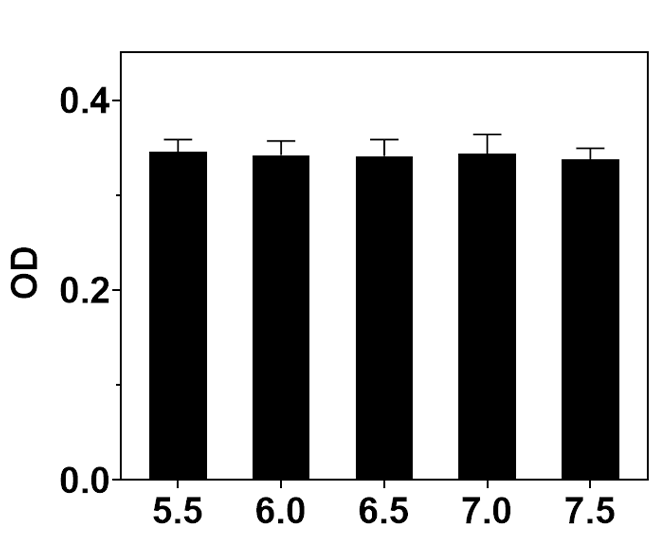


**Figure S4**. Optical Density (OD) measurement of GUL@LsiYY1@MZ dispersed in aqueous solutions with different pH values.


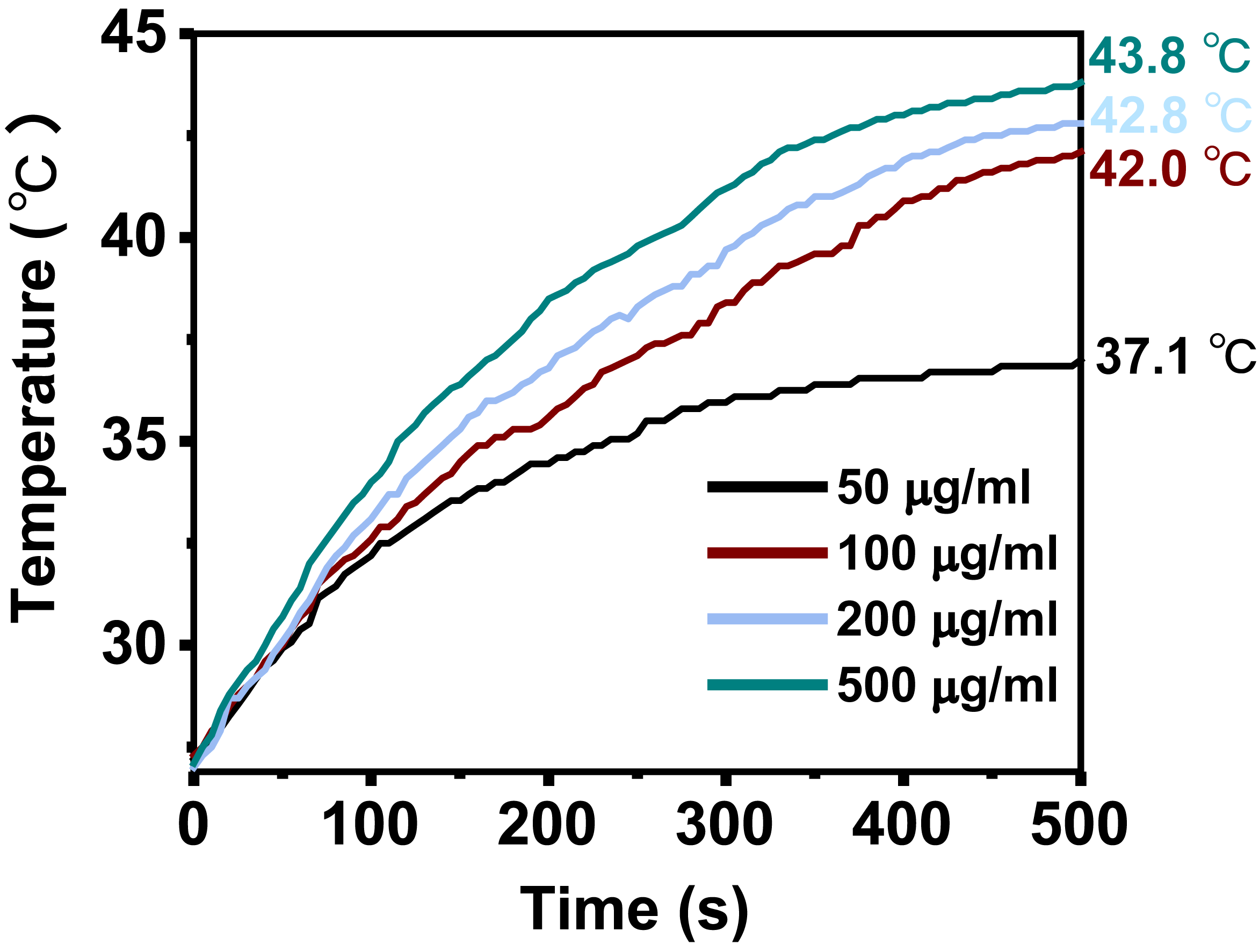


**Figure S5**. Magnetothermal performance of GUL@LsiYY1@ MZ at different concentrations (50, 100, 200, and 500 µg/ml) under AMF for 500 s temperature curves.

**Figure S6**. Temperature-responsive siRNA release from GUL@LsiYY1@MZ in aqueous solution under varying external temperature stimuli.


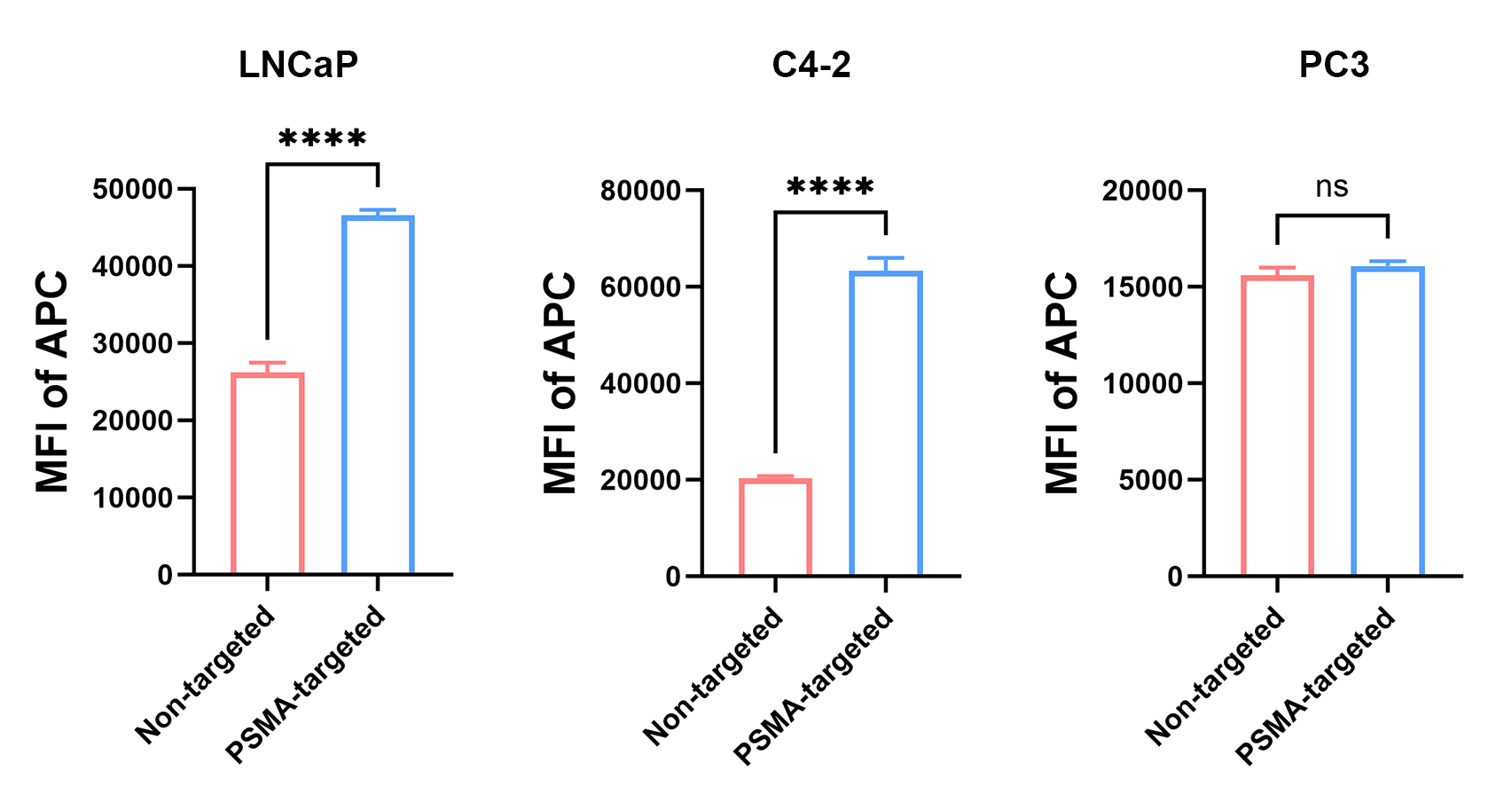


**Figure S7**. The mean fluorescence intensities (MFI) of Cy5-labeled PSMA-targeted and non-targeted nanoparticles in LNCaP, C4-2 and PC3 cells. Data are reported as mean values ± SD (n = 3). The Student's t-test was employed for comparisons between two groups. Significance levels: n.s, not significant; *p < 0.05; **p < 0.01; ***p < 0.001; ****p < 0.0001.


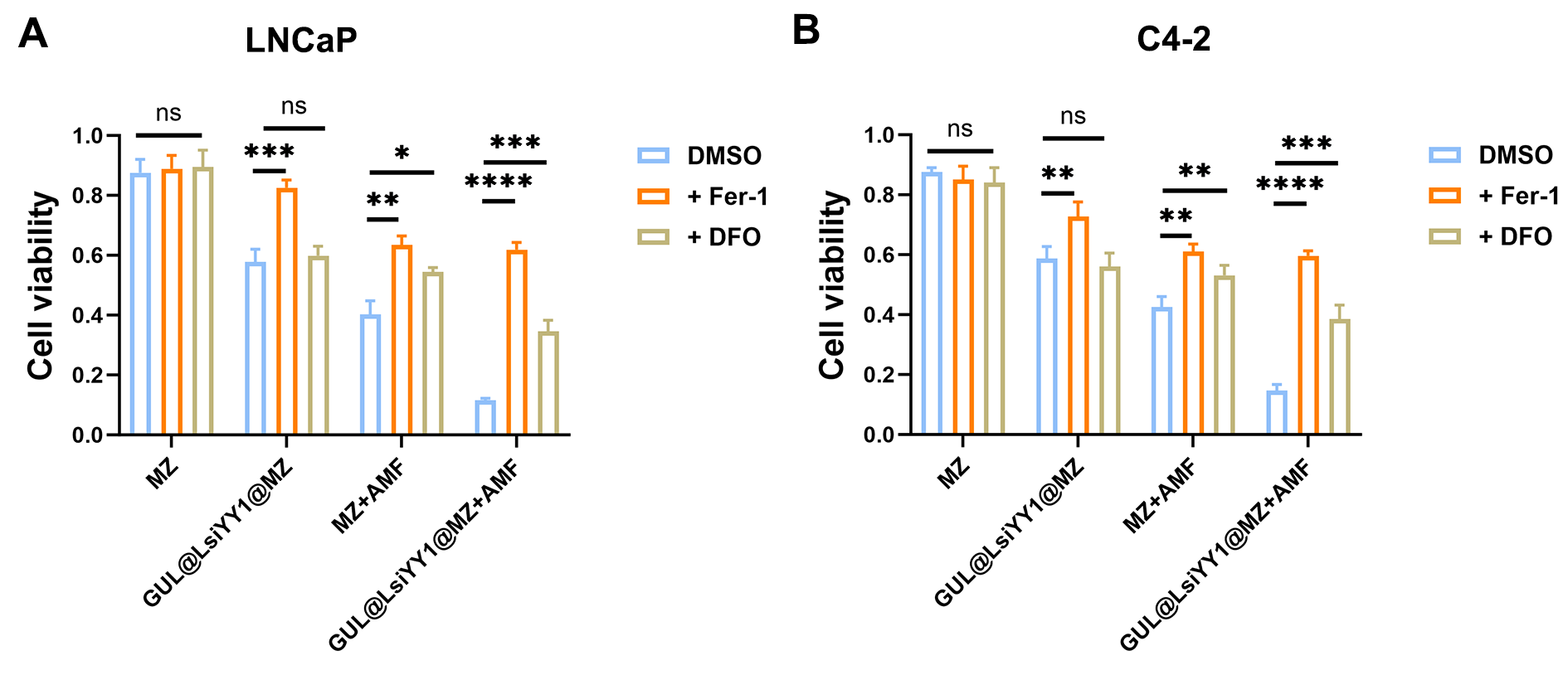


**Figure S8**. Viability of LNCaP and C4-2 cells under different treatments at a concentration of 100 μg/mL for 48 h in the presence and absence of Fer-1 (1 μM) or DFO (100 μM) by CCK-8 assays (n = 5). One-way ANOVA with Tukey’s post-hoc test was used for multi-group comparisons. Significance levels: *p < 0.05, **p < 0.01, ***p < 0.001, ****p < 0.0001.


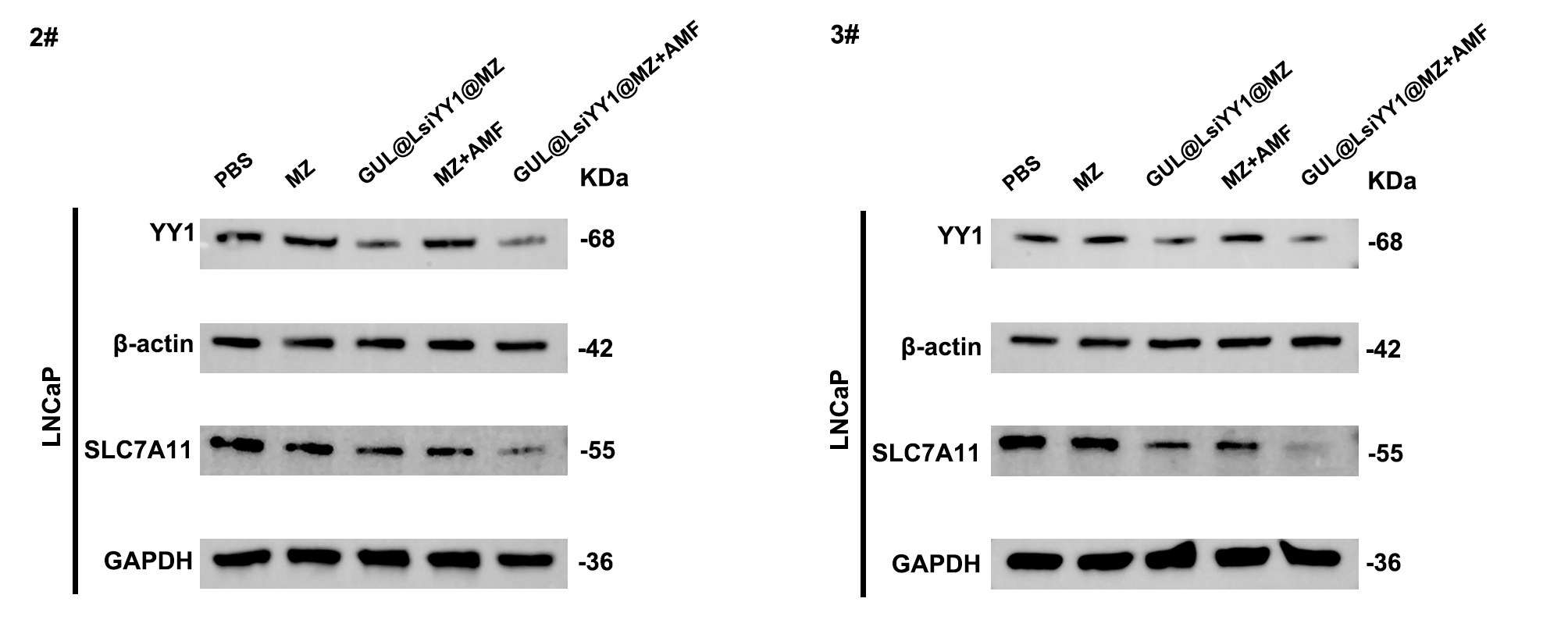


**Figure S9**. Western blotting assays showed the protein level of YY1 and SLC7A11 in LNCaP cell after different treatments.


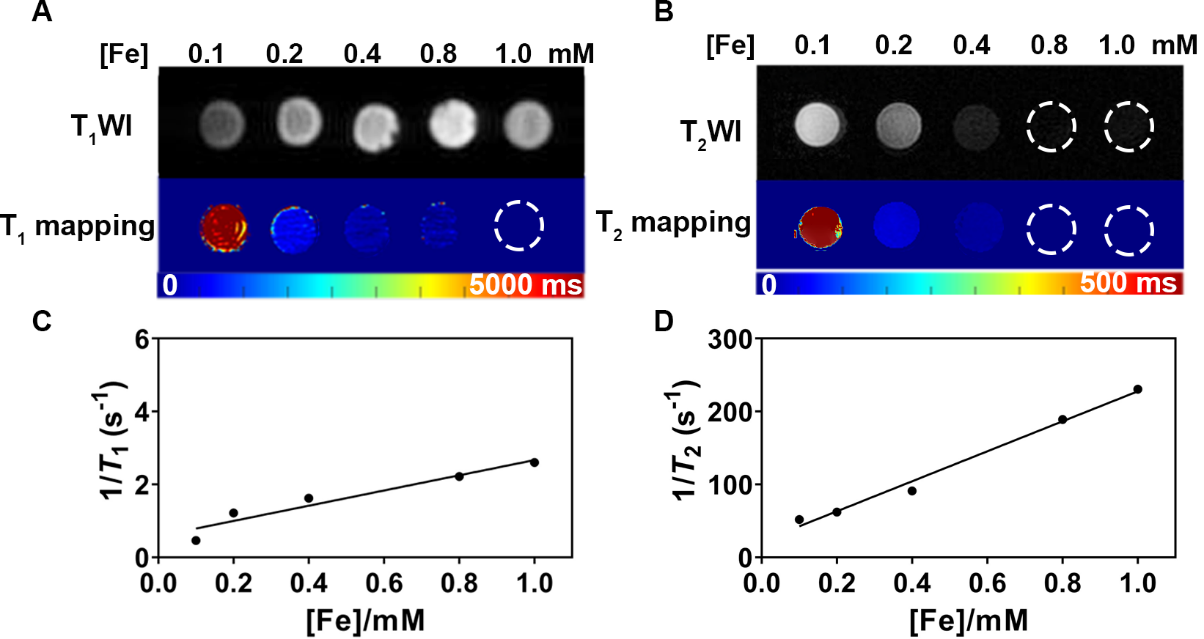


**Figure S10**. MRI properties of GUL@LsiYY1@MZ at various iron concentrations. **(a)** T_1_-weighted MR images (SE sequence) and T_1_ mapping (T_1_ relaxation time was measured using SE-IR sequence with increasing inversion times (TI = 50, 400, 1100 and 2500 ms), and the T_1_-mapping images was generated, the pseudo-color scale corresponds to T_1_ times ranging from 0 to 5000 ms). **(b)** T_2_-weighted MR images (SE sequence) and T_2_ mapping with a multi-echo spin-echo sequence. **(c)** T_1_ relaxation time of GUL@LsiYY1@MZ. **(d)** T_2_ relaxation time of GUL@LsiYY1@MZ.

**Table S1**. Detailed reaction parameters for the synthesis of ultrasmall Mn_x_Zn_1–x_F nanoparticles

| **Samples** | Fe(acac)_3_ (g) | Mn(acac)_2_ (g) | Zn(acac)_2_ (g) |
| --- | --- | --- | --- |
| Mn_0.1_Zn_0.9_F | 0.7064 | 0.0253 | 0.2367 |
| Mn_0.2_Zn_0.8_F | 0.7064 | 0.0506 | 0.2104 |
| Mn_0.4_Zn_0.6_F | 0.7064 | 0.1012 | 0.1578 |
| Mn_0.6_Zn_0.4_F | 0.7064 | 0.1518 | 0.1052 |
| Mn_0.7_Zn_0.3_F | 0.7064 | 0.1771 | 0.0789 |
| Mn_0.8_Zn_0.2_F | 0.7064 | 0.2024 | 0.0526 |
